# Recurrent Domestication by Lepidoptera of Genes from Their Parasites Mediated by Bracoviruses

**DOI:** 10.1371/journal.pgen.1005470

**Published:** 2015-09-17

**Authors:** Laila Gasmi, Helene Boulain, Jeremy Gauthier, Aurelie Hua-Van, Karine Musset, Agata K. Jakubowska, Jean-Marc Aury, Anne-Nathalie Volkoff, Elisabeth Huguet, Salvador Herrero, Jean-Michel Drezen

**Affiliations:** 1 Department of Genetics, Universitat de València, Burjassot, Spain; 2 Estructura de Recerca Interdisciplinar en Biotecnologia i Biomedicina (ERI BIOTECMED), Universitat de València, Burjassot, Spain; 3 Institut de Recherche sur la Biologie de l'Insecte, UMR CNRS 7261, UFR des Sciences et Techniques, Université François Rabelais, Tours, France; 4 Laboratoire Evolution, Génomes, Comportement, Ecologie, CNRS/Université Paris-Sud UMR9191, IRD UMR247, Université Paris-Saclay, Gif-sur-Yvette, France; 5 Commissariat à l’Energie Atomique et aux Energies Alternatives, Genoscope (Centre National de Séquençage), Evry, France; 6 Diversity, Genomes and Interactions Between Microorganisms and Insects Laboratory, INRA (UMR 1333), Université de Montpellier, Place Eugène Bataillon, CC 101, Montpellier, France; University of Utah School of Medicine, UNITED STATES

## Abstract

Bracoviruses are symbiotic viruses associated with tens of thousands of species of parasitic wasps that develop within the body of lepidopteran hosts and that collectively parasitize caterpillars of virtually every lepidopteran species. Viral particles are produced in the wasp ovaries and injected into host larvae with the wasp eggs. Once in the host body, the viral DNA circles enclosed in the particles integrate into lepidopteran host cell DNA. Here we show that bracovirus DNA sequences have been inserted repeatedly into lepidopteran genomes, indicating this viral DNA can also enter germline cells. The original mode of Horizontal Gene Transfer (HGT) unveiled here is based on the integrative properties of an endogenous virus that has evolved as a gene transfer agent within parasitic wasp genomes for ≈100 million years. Among the bracovirus genes thus transferred, a phylogenetic analysis indicated that those encoding C-type-lectins most likely originated from the wasp gene set, showing that a bracovirus-mediated gene flux exists between the 2 insect orders Hymenoptera and Lepidoptera. Furthermore, the acquisition of bracovirus sequences that can be expressed by Lepidoptera has resulted in the domestication of several genes that could result in adaptive advantages for the host. Indeed, functional analyses suggest that two of the acquired genes could have a protective role against a common pathogen in the field, baculovirus. From these results, we hypothesize that bracovirus-mediated HGT has played an important role in the evolutionary arms race between Lepidoptera and their pathogens.

## Author Summary

Eukaryotes are generally thought to evolve mainly through the modification of existing genetic information. However, evidence of horizontal gene transfer (HGT) in eukaryotes-the accidental acquisition of a novel gene from another species, allowing acquisition of novel traits—is now recognized as an important factor in their evolution. We show here that in several lineages, lepidopteran genomes have acquired genes from a bracovirus that is symbiotically used by parasitic wasps to inhibit caterpillar host immune defences. Integration of parts of the viral genome into host caterpillar DNA strongly suggests that integration can sporadically occur in the germline, leading to the production of lepidopteran lineages that harbor bracovirus sequences. Moreover, some of the transferred bracovirus genes reported here originate from the wasp genome, demonstrating that a gene flux exists between the two insect orders Hymenoptera and Lepidoptera that diverged ≈300 MYA. As bracovirus gene organisation has evolved to allow expression in Lepidoptera, these transferred genes can be readily domesticated. Additionally, we present functional analyses suggesting that some of the acquired genes confer to caterpillars a protection toward baculovirus, a very common pathogen in the field. This phenomenon may have implications for understanding how caterpillars acquire resistance against baculoviruses used in biological control.

## Introduction

Unlike bacteria, which have obtained a notable proportion of their genes through the acquisition of sequences from distantly related organisms, eukaryotes are generally thought to evolve mainly through the modification of existing genetic information [1]. However evidence of horizontal gene transfer (HGT) in eukaryotes is accumulating and is recognized as an important factor in their evolution and acquisition of novel traits [2–5]. The majority of events reported concerns transposable elements, DNA sequences capable of excising or copying themselves from one genomic locus to integrate into another locus [6]. Genome sequencing has revealed that eukaryotes have also acquired DNA from symbionts and parasites, probably because the intimacy of these relationships favours DNA exchange. For example, numerous insect and nematode genomes contain sequences originating from Wolbachia [7, 8] an endocellular bacteria widespread in insect populations infecting, in particular, host germ line cells [9]. Recently, a systematic investigation of HGT events in three available lepidopteran genomes (*Bombyx mori*, *Danaus plexippus* and *Heliconius melpomene)* revealed multiple ancient HGT events from bacteria and fungi to these lepidopteran genomes [10]. Here we present an original mode of HGT between two insect orders based on the integrative properties of a virus (bracovirus) that has evolved within parasitic wasp genomes for ≈100 million years and that is used to facilitate the development of their progeny in caterpillars by inhibiting host immune defenses. In one case, we could demonstrate the direction of the transfer based on the presence of a sequence important for the virus life cycle. This is a rare example where the likely mechanism of HGT can be established in an animal system. Moreover we present functional analyses suggesting that some of the transferred genes have been recycled by Lepidoptera to protect them against a common viral pathogen.

Bracoviruses play a central role in parasite-host interactions involving parasitic wasps and their caterpillar hosts. Bracoviruses are injected by parasitic wasps into their hosts along with wasp eggs. These wasps develop during their larval stage within the body of their lepidopteran hosts. Tens of thousands of species of wasps belonging to the braconid family and parasitizing a large diversity of lepidopteran species are each associated with a specific bracovirus [11]. All these associations originated from a single integration event of a nudivirus genome in a common ancestor of the wasps [12]. Since this integration ≈100 MYA, the genes involved in virus particle production have been dispersed in the wasp genome, they are no longer packaged in the particles that contain genes encoding virulence factors. Moreover the endogenous chromosomally transmitted virus has evolved depending on its contribution to parasitism success, resulting in a specific set of virulence genes packaged in the particles in the different wasp lineages [13]. These viruses are now essential for successful development of the wasp larvae within lepidopteran hosts [13–15]. Viral replication and particle production occur exclusively in the wasp ovaries from endogenous viral elements present in the wasp genome. The particles, that contain dsDNA circles harbouring the virulence genes, constitute the major component of the fluid injected with the eggs into the parasitized caterpillar host during wasp oviposition. Once in the host body the particles enter lepidopteran host cells and the host cellular machinery expresses these virulence genes. Viral products ensure wasp larvae survival in the lepidopteran body by interfering with caterpillar host immune responses and development [16, 17].

The dsDNA circles packaged in the particles are produced from chromosomally transmitted proviral segments stably integrated in the wasp genome [18–21]. The typical eukaryotic organization of the genes transferred by the particles [22] and their lack of similarity with viral genes suggest they originate from the wasp genome, which could be demonstrated for a few of them by phylogenetic analyses [23]. However many genes have diverged in their sequence from insect genes, to the extent that they are currently no more closely related to wasp genes than to mammalian genes [24, 25]. Many other bracovirus genes have unknown origins and display no similarities to genes in data banks except with other bracovirus sequences. For example, Cotesia congregata bracovirus (CcBV) encodes 26 bracovirus specific gene families (named BV1 to BV26)[18].

We previously reported that some viral circles were found to be reintegrated in the genome of different geographic strains of the wasp *Cotesia sesamiae* [13, 26]. The occurrence of circle integrations back into wasp genomes probably reflects a broad integration ability of circles since it was recently shown that integration into the DNA of parasitized lepidopteran host cells is a part of the bracovirus life cycle. Indeed it was shown that Microplitis demolitor bracovirus circle integration into lepidopteran *Pseudoplusia includens* DNA occurs by a specific mechanism involving a conserved viral site named Host Integration Motif [27]. During integration the circles are opened specifically at this site, resulting in integrated forms readily distinguishable from that of the proviral form [27]. The analysis of Cotesia sesamiae bracovirus (CsBV) reintegrated circles suggests that the same mechanism was involved in their integration back into the wasp genome [13, 26].

Parasitized caterpillars represent most of the time an evolutionary dead-end since parasitoid wasps inhibit metamorphosis [28] and the host usually does not survive parasitism [29]. However it is conceivable that some hosts might successfully defend themselves against the parasite by interrupting wasp oviposition, eliminating the eggs or killing the larvae, resulting in the reproduction of Lepidoptera that have been infected by bracoviruses. Parasitoid wasps could also target non-host species and fail to interfere with their development [30]. We can speculate that caterpillar escape from the fatal issue after virus injection could allow bracovirus particle entry into germ cells and in rare cases stable integration of circles into lepidopteran genomes.

To determine if bracovirus sequences could indeed be integrated into lepidopteran genomes, we compared the DNA sequences packaged in the particles of Cotesia congregata bracovirus (CcBV), the genome of which is almost completely characterized [18], to a series of genomes from non-host Lepidoptera of the parasitoid wasp *C*. *congregata* and from *Manduca sexta*, a regular host. Here, in contrast to a previous bioinformatic analysis listing a series of bracovirus insertions, most of them relatively short [31], we searched for large nucleotide stretches (more than 500 bp long) that could encode potentially domesticated genes by lepidopteran species and evaluated the evolutionary meaning of these integrations by functional analysis of two of the transferred genes. Similarity searches allowed the identification of bracovirus DNA insertions in the genomes of the monarch (*Danaus plexippus*), the silkworm (*Bombyx mori*), the beet armyworm (*Spodoptera exigua*) and the fall armyworm (*Spodoptera frugiperda*) but not in the genome of tobacco hornworm (*M*. *sexta*), the regular host of *Cotesia congregata*. All these insertions were characterized by the presence of large stretches of nucleotide sequences strikingly similar to those of bracoviruses (close to 90% identities at the nucleotide level) flanked by lepidopteran-specific sequences. Insertions include genes but also in some cases parts of bracovirus circles, the organization of which has been conserved, indicating the direction of HGT was from bracovirus to Lepidoptera. Moreover, in one insertion a regulatory signal involved in dsDNA circle production in the wasp has been retained, constituting an unambiguous signature of the bracoviral origin of the sequence since bracovirus replication is non autonomous and occurs exclusively in the wasp ovaries.

Altogether our data indicate that bracoviruses have been a recurrent source of genes for Lepidoptera. Moreover functional analyses provide convergent results suggesting that some of these genes might contribute to protect larvae against baculoviruses, deadly pathogens that threaten them in the field, which would explain why they have been maintained in lepidopteran genomes.

## Results

### Bracovirus sequences encoding BEN proteins in the monarch and silkworm genomes

#### Presence and molecular evolution of Bracovirus-like Ben sequences in the monarch genome

**Fig 1:**
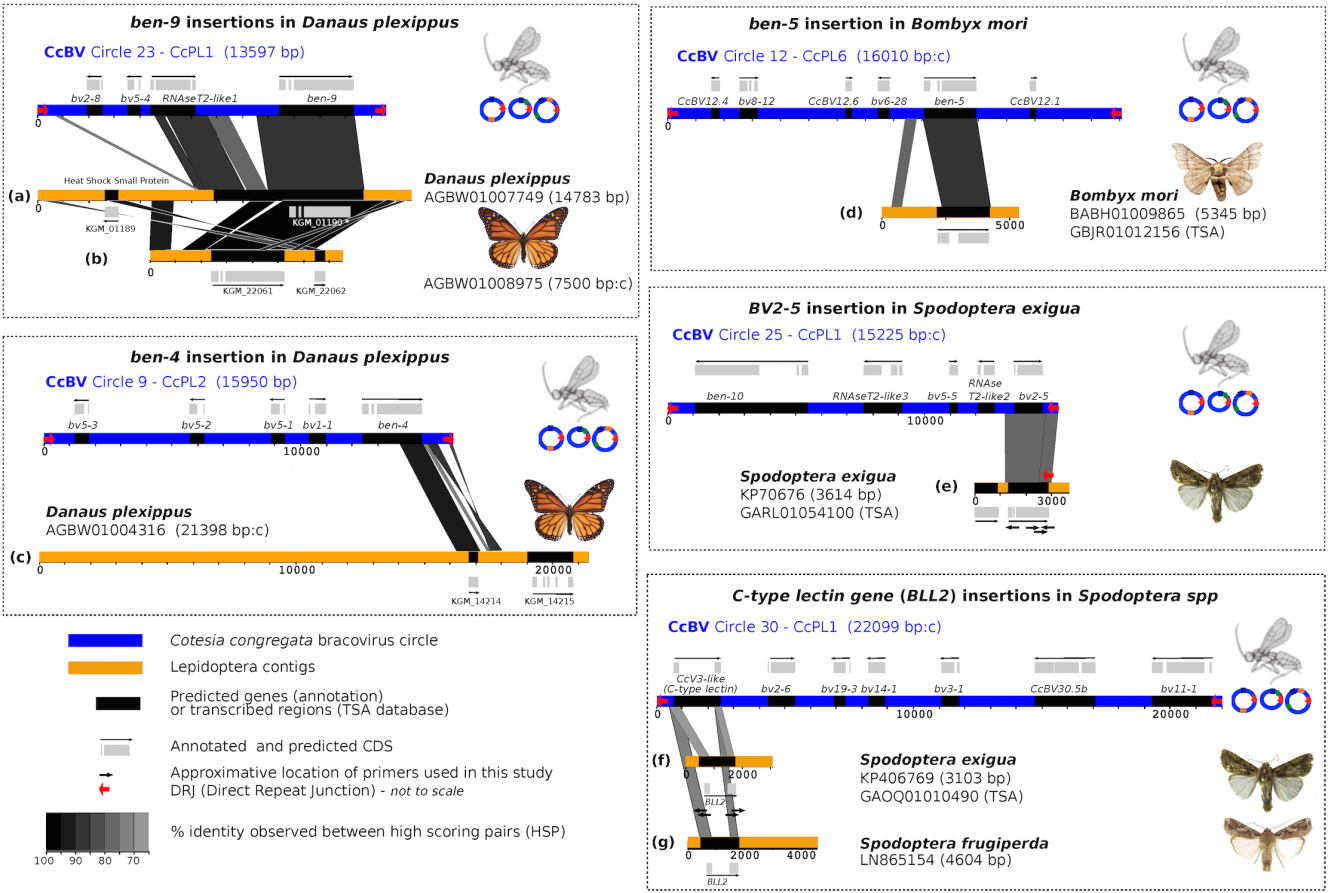
Map of bracovirus sequences inserted into lepidopteran genomes. The seven examples of high homology regions between lepidopteran sequences and bracovirus circles (CcBV) described in this paper are shown (a to g correspond to the different insertions of bracovirus sequences related to CcBV found in Lepidoptera genomes). The level of similarity is indicated by grey colour intensity. Sequences of Lepidoptera contigs flanking the homology regions correspond to lepidopteran genomic DNA identified as such by specific genes and/or repetitive sequences of lepidopteran genomes. CcBV sequences are shown as in their integrated proviral form in the wasp genome in direct orientation or reverse complement (indicated by a c after contig length). Position of primers used to extend sequences or to verify insertions in different species or to check for splicing are shown. Gene annotations (reported from Genbank) and detected transcripts (TSA) are indicated.

Three sequences highly similar to CcBV (> = 88% at the nucleotide level) were identified within the monarch (*Danaus plexippus*) genome, with two insertions highly similar to CcBV Circle 23 (C23) sequence (6560 bp and 4075 bp long, Fig 1(A) and 1(B) respectively) and one insertion highly similar to CcBV Circle 9 (C9) (1768 bp long: Fig 1(C)). The two monarch genomic regions with C23-like insertions correspond most likely to a duplication after a single ancestral integration event, since they are highly similar (>97%) and similarities extend into flanking lepidopteran sequences with the presence of a *heat shock protein* gene, albeit in a different position indicating rearrangements have occurred (Fig 1(A) and 1(B)). Notably the organization of the largest insertion (Fig 1(A)) is strikingly similar to that of the bracovirus sequence with two CDS coding for *RnaseT2* and *Ben9* genes, separated by non-coding sequences homologous to those found between the two genes in C23 (Fig 1). The C9 insertion (Fig 1(C)) contains a truncated version of a different *Ben* gene (*Ben4*, Fig 1) encoding the C-terminal part comprising the BEN domain.

**Fig 2:**
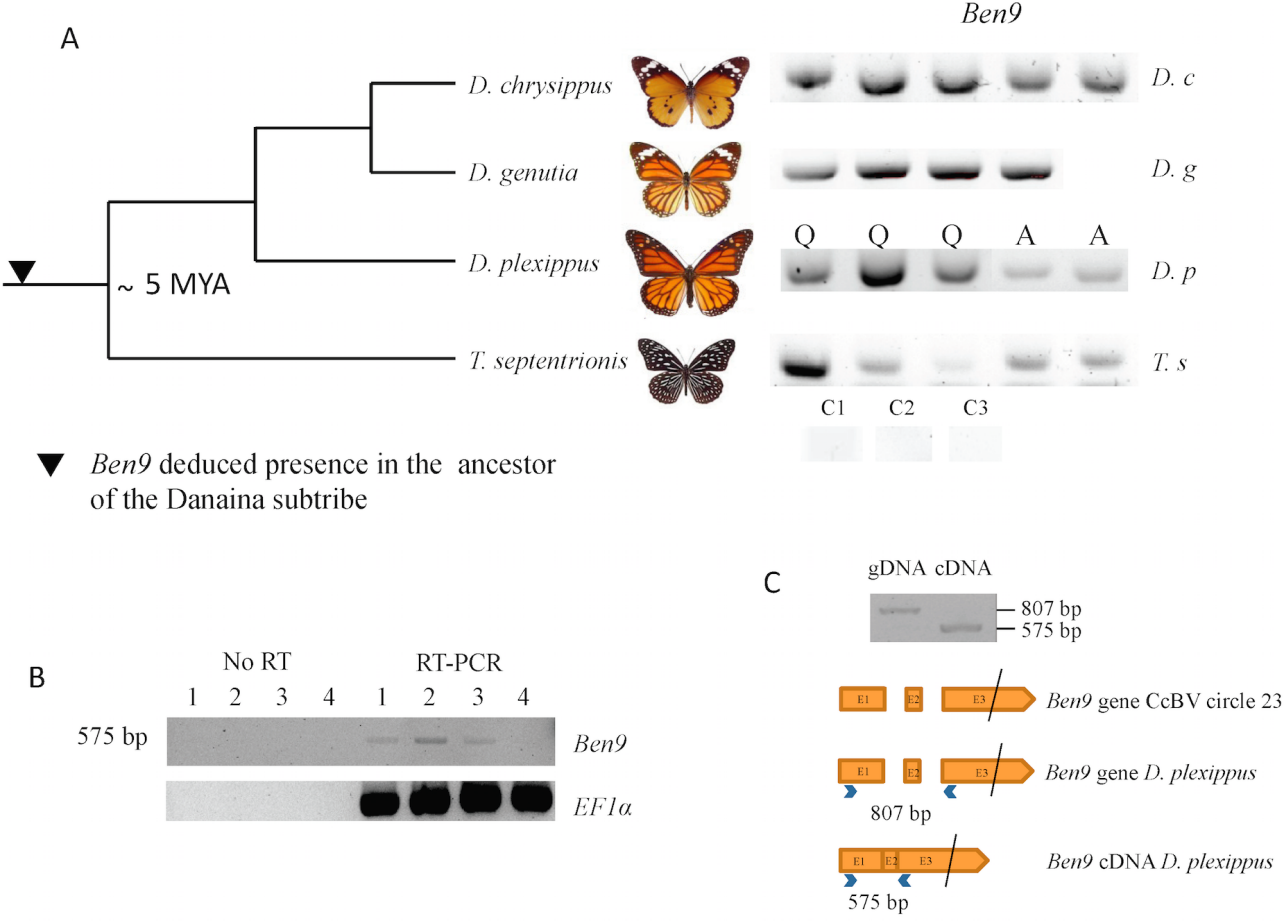
Analysis of BEN 9 encoding insertions in the *Danaina* subtribe. A) Analysis of BEN9 encoding insertions in genomic DNA of individuals from different species of the *Danaina* subtribe by *ben9* gene PCR amplification from Lepidoptera of the species *Danaus chrysippus chrysippus* (Oman), *Danaus genutia* (Thailand), *Danaus plexippus* (Q, caterpillar sampled in Québec, A, adults from Australia), *Tirumala septentrionis septentrionis* (Malaysia). C1, C2, C3: control PCR (without DNA) performed with primer pairs used respectively for *D*. *plexippus*, *D*. *chrysippus*/*D*. *genutia* and *T*. *septentrionis* PCRs B) RT-PCR analysis of *Ben9* expression in *D*. *plexippus* caterpillars from Québec. *Ben9* expression was detected in three individuals. No PCR amplification of *Ben9* was observed on RNA samples that were not subjected to RT (No RT). C) PCR fragments obtained from *D*. *plexippus* genomic DNA and cDNA and schematic represention of *Ben9* gene and *D*. *plexippus Ben9* cDNA organization. The black bar indicates that exon 3 is not to scale. Note that in the amplified fragment corresponding to *D*. *plexippus* cDNA, the two *Ben9* intron sequences have been excised as observed in *Ben9* cDNA obtained from *Manduca sexta* parasitized by *Cotesia congregata* [33]. The phylogenetic tree is adapted from [34]. Dating of the common ancestor is reported from [35].

Since the *Danaus plexippus* genome was initially sequenced from only three individual butterflies [32] we verified that the insertions were common in the monarch by assessing the presence of bracovirus-like sequences in the DNA of 5 individuals collected from new geographic locations (Canada and Australia). PCR and sequencing confirmed that *Ben9* (Fig 2A) and *Ben4* (S1 Fig) bracovirus related sequences were present in all the individuals tested, suggesting these insertions are probably fixed in the species.

**Fig 3:**
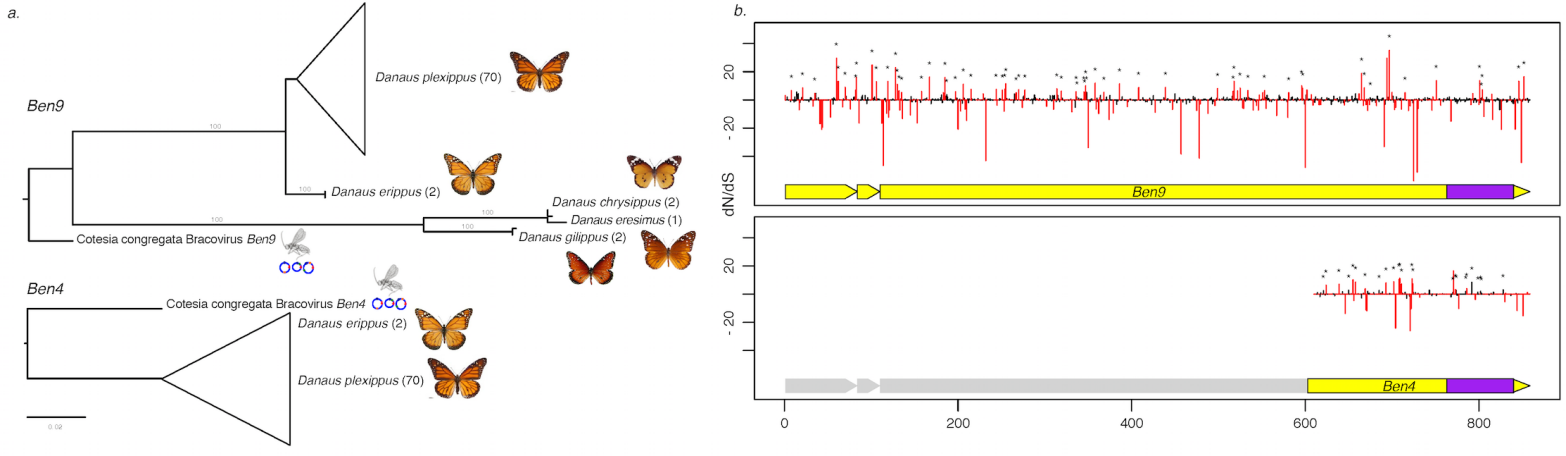
Measure of selection operating on *Ben4* and *Ben9* genes in *D*. *plexippus* and 4 related species. a) Phylogenetic tree based on the nucleotide sequence alignment of the region shared between *Ben4* and *Ben9* in *Danaus* species samples and CcBV. The values in brackets indicate the number of lepidopteran individuals used in the analysis. b) Plot of the dN/dS value of each codon along the *Ben* genes based on the alignment of the butterfly sequences. The red bars represent values that are significantly under positive or negative selection (HyPhy, p-value ≤ 0.1). The asterisks identify the sites also under positive selection with the PAML approach. The yellow blocks under the dN/dS graphs represent the *Ben* gene structure composed of three exons. The first exon corresponds to a PHA02737 domain and the BEN domain (represented in purple) is encoded by the end of the third exon. Note that the truncated *Ben4* gene conserved in *D*. *plexippus* corresponds to the third exon of CcBV *Ben4* gene, which contains the BEN domain.

We could further confirm the presence of the two *Ben9* and *Ben4* gene insertions in the genome of the monarch and 4 related species by analysing the data obtained (Illumina sequences) from 88 individuals recently used to study the relationship between monarch populations and their migration patterns [30]. For all individuals we could identify the genes corresponding to the *Ben4* (Fig 1(C)) and the two *Ben9* insertions (Fig 1(A) and 1(B)) by mapping reads onto the monarch reference genome (monarch individuals) or by *de novo* assembly (individuals from related species). We found that three gene copies encoded truncated *Ben4* and *Ben9* proteins (in eight and twelve individuals respectively) but the reading frames downstream of the stop codons were still identifiable, indicating relatively recent mutation events. We checked for molecular signatures associated with particular selection that may act on *Ben4* and *Ben9* genes, by measuring ratios of non-synonymous versus synonymous substitutions on all gene copies (including those with a stop codon). The global dN/dS in each gene showed a moderate level of purifying selection (ω <1) with both HyPhy and PAML (*Ben4*: ω = 0.80585 and ω = 0.90717, *Ben9*: ω = 0.69803 an ω = 0.80628 with HyPhy and PAML respectively) but this level could be affected by the presence of non-functional gene copies (including those with a stop codon). We also measured the dN/dS ratio for each codon in the genes. We observed mostly sites under purifying selection (55.6% of sites with Hyphy and 68.4% with PAML in *Ben4* and 39.8% of sites with Hyphy and 74.2% with PAML in *Ben9*, for a total of 250 and 852 sites respectively) and a few sites under positive selection (6.0% of sites with Hyphy and 10.7% with PAML in *Ben4* and 4.3% of sites with Hyphy and 8.6% with PAML in *Ben9*); most of the sites found to be under positive selection with Hyphy were also detected to be under positive selection with PAML (Fig 3). The other positions (38.4% of sites with Hyphy and 20.9% with PAML in *Ben4* and 55.9% of sites with Hyphy and 17.2% with PAML in *Ben9*) evolved neutrally or are affected by pressures too weak to be detected. These results are compatible with genes having a function in the monarch (Fig 3) and coding for proteins that interact with targets that can be modified by host/pathogen arms race. However since knowledge on BEN proteins is limited it is difficult to relate selection on particular sites with the function of the protein.

#### Ancestry of bracovirus-like BEN encoding sequences in the Danaina subtribe and genomic organization of insertions

The presence of bracovirus sequences in the monarch is however unexpected considering that this species is not reported among the hosts of bracovirus-associated wasps (J. Whitfield personal communication). To assess whether these insertions could be ancient in the monarch lineage, we performed PCRs and sequencing of amplified fragments using DNA extracted from individuals of a series of phylogenetically related species. We could thus trace back the presence of bracovirus *Ben9* sequences in the common ancestor of the genus *Danaus* and its sister genus *Tirumala* composing the subtribe *Danaina* [34, 36] that lived ≈5 million years ago [35], indicating that the insertions were ancient (Fig 2A).

#### Presence of Bracovirus-like Ben sequences in the silkworm genome

A *Ben 5* gene-encoding region was also detected in the genome of the silkworm with two sequences highly similar to CcBV C12 (411 bp and 1916 bp long sharing 80% and 88% nucleotide sequence similarity with CcBV C12 respectively) separated by a lepidopteran sequence (1515 bp long)(Fig 1(E)). We did not study further this HGT since the presence of this bracovirus sequence in the silkworm was confirmed experimentally in a recent study [31].

#### Ben9 is expressed in monarch larvae

The frequency of BEN-encoding insertions might be related to the fact that the *Ben* gene family, containing 14 genes, is the second most abundant in CcBV. The complete predicted BEN proteins are the largest bracovirus proteins, with up to 1200 amino acids. The N-terminal region contains a conserved domain (PHA02737: 68 amino acids) and the C-terminal region corresponds to the BEN domain (approx. 100 amino acids). This BEN domain was first defined by computational analyses as a conserved α-helical module present in diverse animal proteins and in viruses from two unrelated families, chordopoxviruses and bracoviruses [37] and was predicted to mediate protein-DNA and protein-protein interactions during chromatin organization and transcription [37]. More recently, *C*. *vestalis* BEN9 was reported to induce host immune suppression, based on a functional analysis using purified bracovirus segment injection coupled with RNA interference [38] while in *Drosophila* a BEN domain containing protein was shown to bind to specific DNA sequences and act as a transcriptional repressor [39].

Here, expression of the *Ben9* gene (but not *RnaseT2* gene) was detected by RT-PCR in three out of four *D*. *plexippus* larvae tested. Furthermore, the two predicted introns were spliced, suggesting a BEN9 protein is potentially produced and functional in the monarch (Fig 2C). The fact that several *Ben* genes have been maintained in lepidopteran genomes, that *Ben9* gene is expressed in the monarch and that the selection operating on *Ben9 and Ben4* is mostly conservative suggests the function of these proteins might be useful for Lepidoptera.

### Bracovirus sequences encoding BV2-5 and lectins in *Spodoptera* genomes

#### Bracovirus sequences encoding BV2-5 in *Spodoptera* spp.

Based on a detailed analysis of the larval transcriptome of the Lepidoptera *S*. *exigua* [40] we also revealed the presence of seven sequences highly similar to those of bracoviruses. One sequence contained an insertion (1548 bp long) highly similar to CcBV C25 (90% sequence identity at the nucleotide level, Fig 1(E)). This sequence encodes *BV2-5* a member of a bracovirus-specific BV gene family (BV-2), which comprises 8 genes in CcBV. *BV2-5* is among the most highly expressed genes in the parasitized host (*Manduca sexta*) fat body and haemocytes [22]. The intron present in the CcBV gene version [22] is also spliced in the *S*. *exigua BV2-5* homologue (Fig 1, annotated CDS) indicating that the *BV2-5* gene organization has been conserved and the splice signals are recognized in these two families of Lepidoptera (Sphingidae and Noctuidae). The *BV2-5* gene was found in *S*. *exigua* DNA from different geographic sources (America, Europe, Japan). However, European populations contained a frame shift mutation generating a truncated form of the protein (S2 Fig). Interestingly, data mining indicated that *BV2-5* homologues are also expressed in *S*. *littoralis* (H. Vogel personal communication) and *S*. *litura* (Genbank).

#### Bracovirus sequences encoding lectins in *Spodoptera* spp. and evidence of bracovirus-mediated gene flux between Hymenoptera and Lepidoptera.

**Fig 4:**
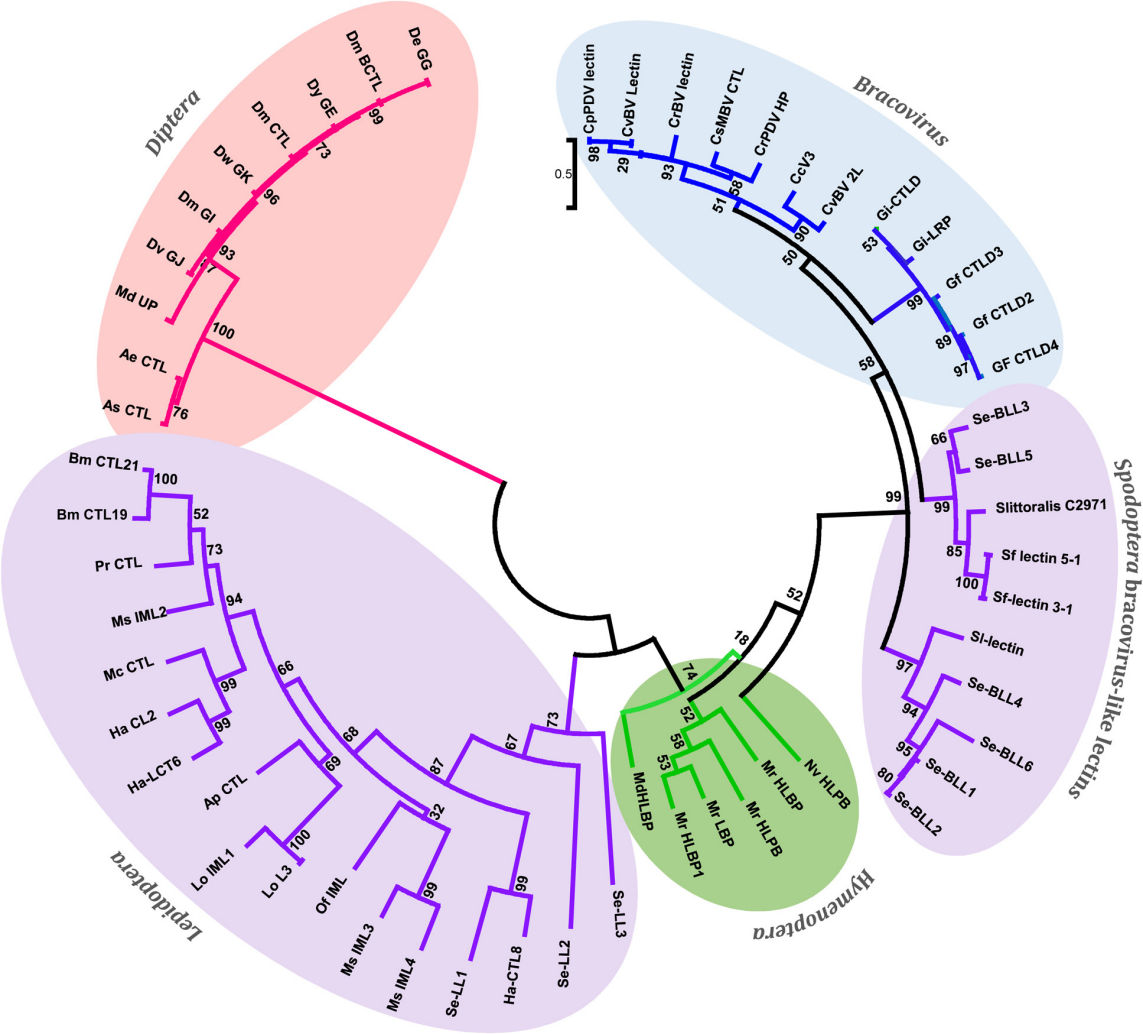
Phylogenetic tree of bracovirus-lectin like proteins from different *Spodoptera* species and their homologs from bracovirus, hymenopteran, lepidopteran and dipteran species. Evolutionary distance was calculated for aligned sequences by Maximum likelihood analysis. The tree was performed using the alignment of the C-lectin domains of the proteins analysed (S3 Fig).

A second group of *S*. *exigua* ESTs is composed of 6 sequences (500 bp long) with significant similarity to the C-type lectin gene of bracoviruses (close to 70% nucleotide identities). Accordingly we named these genes *S*. *exigua* bracovirus-like lectin genes (Se-*BLLs*). All 6 Se-*BLLs* code for hypothetical proteins of about 20 kDa with a predicted signal peptide at their N-terminus. Closely related sequences were also found by data mining in *S*. *furgiperda*, *S*. *littoralis* and *S*. *litura* (named BLLs). Since the homology between bracovirus and Lepidoptera sequences is less striking than for *Ben9* and *BV2-5* insertions, we performed alignments (S2 Fig) and phylogenetic analysis using distance and maximum likelihood to verify the relationships of the predicted products with bracovirus proteins. The results obtained with the two methods were similar and clearly showed that BLLs share a common history with bracovirus and hymenopteran lectins and that they are more distantly related with other C-type lectins from Lepidoptera (Fig 4).

Since C-type-lectins are not present in all bracoviruses but only in a specific lineage of the microgastrinae subfamily (including *Cotesia* and *Glyptapanteles* species but not *Microplitis demolitor*) the phylogenetic analysis strongly suggests an evolutionary scenario in which a C-type-lectin was transferred from the wasp genome to the proviral form of the bracovirus in an ancestor of this lineage resulting in its incorporation in a bracovirus circle (as shown for a sugar transporter gene in wasp species of the *Glyptapanteles* lineage [23]), later allowing its transfer to the *Spodoptera* lineage. Accordingly Bracovirus C-type-lectins and Se-BLLs belong to a very well supported monophyletic group (bootstrap value = 99), which is clearly related to Hymenoptera C-type-lectins (bootstrap value = 74). Moreover, the fact that Se-BLLs are organized in two clades suggests that two events of bracovirus C-type-lectin gene acquisition followed by gene family expansion occurred in the *Spodoptera* lineage (Fig 4). Altogether C-type-lectin phylogeny indicates that a bracovirus-mediated gene flux exists between the 2 insect orders Hymenoptera and Lepidoptera.

#### Genomic organization of bracovirus-related genes in *Spodoptera* species

To determine the organisation of bracovirus-related genes in *S*. *exigua* genome, genomic sequences flanking *BV2-5* and *Se-BLL2* were isolated by PCR-based DNA walking. We also obtained the integrated form of the Sf 5.1 *C-lectin* gene from the draft genome of *S*. *frugiperda* (Fig 1(G)). We thus confirmed these genes were present in Lepidoptera genomes and are flanked by sequences containing lepidopteran specific repetitive sequences (Fig 1). The other *BLLs* genes are known only by their cDNA sequences.

Fragments of 3687 and 2529 bp were obtained for *BV2-5* and *Se-BLL2*, respectively (Fig 1E and 1F)). The flanking sequence upstream of *BV2-5* contains a retrotranscriptase from a mobile element (jockey-like) found in several lepidopteran species which is expressed in *Spodoptera exigua* (from TSA database Fig 1E), indicating that this part of the fragment corresponds to the lepidopteran genome. The two extremities of the *Se-BLL2* containing fragment also show high nucleotide similarity (70–80%) with sequences from several *Spodoptera* species available in Genbank.

**Fig 5:**
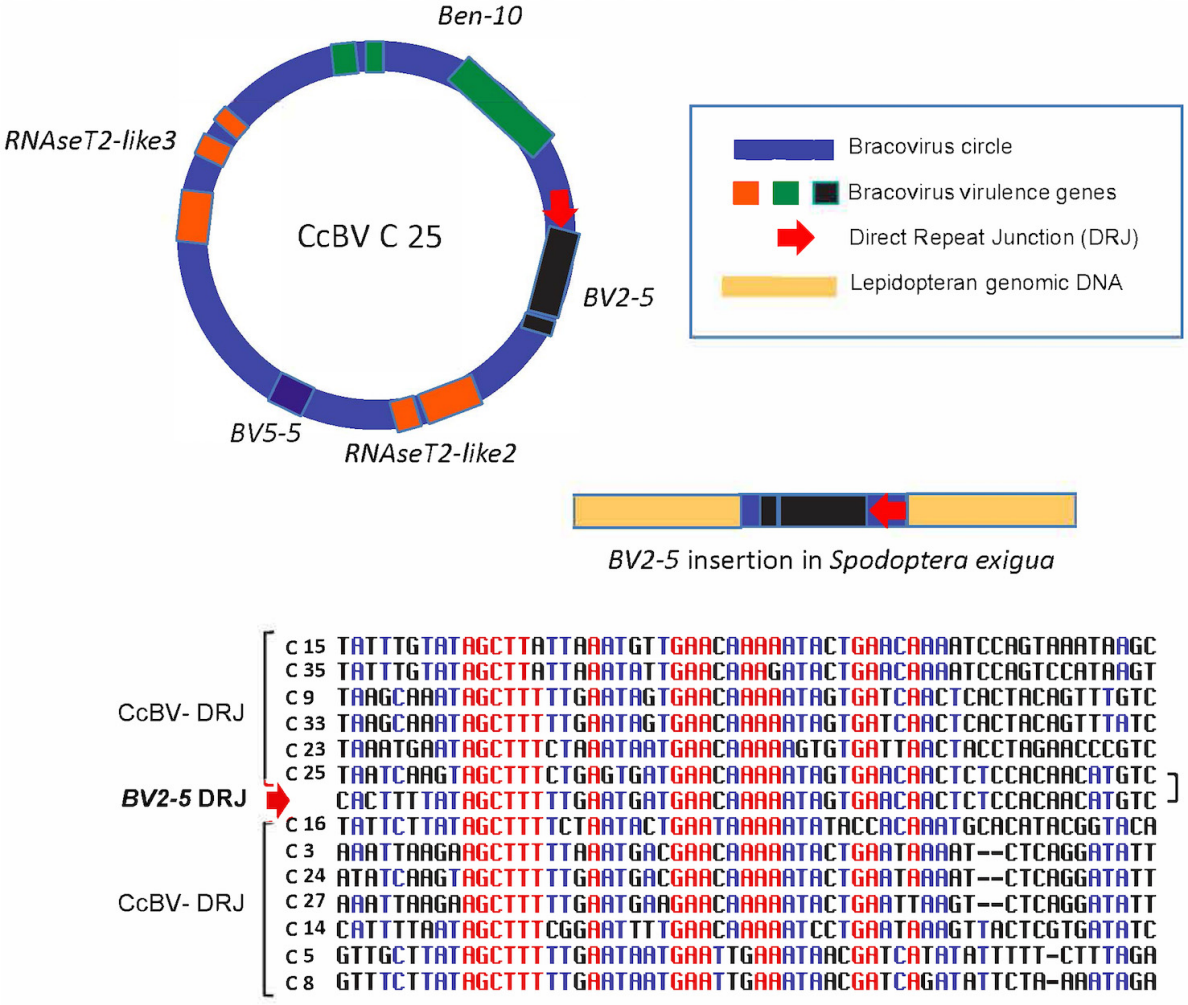
Regulatory sequence involved in bracovirus circle production retained in a bracovirus insertion in *Spodoptera exigua* genome. In the insertion containing *BV2-5*, a sequence (*BV2-5* DRJ) downstream of the gene strongly resembles C25 DRJ of CcBV (C25). A schematic representation of the C25 circle and the BV2-5 insertion in *Spodoptera exigua* genome (not to scale). An alignment of *BV2-5* DRJ with DRJ sequences of 12 CcBV circles (including C25) is shown below. Note that the DRJ in the lepidoptera is in the same relative position as in C25 and that the similarity between C25 DRJ and the BV2-5 insertion extend beyond (residues in black) the most conserved region of the CcBV DRJs (residues in red). The presence of this DRJ sequence, which is important for bracovirus life cycle (production of DNA circles packaged in the particles), is a signature that the sequence originated from a bracovirus and shows that the direction of the transfer was from bracovirus to Lepidoptera.

Analysis of the *BV2-5* fragment revealed the presence of a sequence (2246 bp long) highly similar to CcBV C25, containing sequences both upstream and downstream of *BV2-5* that are present in the bracovirus. The *Se-BLL2* gene fragment contains a *C-type-lectin* gene including the intron (Fig 1F) and a short bracovirus upstream sequence. The *Sf 5*.*1* gene (BLL) organization is similar (Fig 1G). Strikingly the analysis of the *BV2-5* insertion also revealed the presence of a 40 bp regulatory sequence (Direct Repeat Junction, DRJ) involved in bracovirus circle production. This sequence is highly similar to the DRJ of CcBV C25 and is at the same position in the *BV2-5* containing sequence as in C25, which is an unambiguous signature of the bracoviral origin of this sequence (Fig 5). This DRJ was used as a query for blastn analysis (NR data bank) and the retrieved sequences sharing similarity corresponded only to bracoviral DRJs of *Cotesia congregata*. Moreover no sequence was retrieved from WGS data base (NCBI) restricted to Lepidoptera genomes, indicating this DRJ is not generally present within lepidopteran genomes and confirming that this sequence could not be found by chance in *Spodoptera exigua* DNA.

#### Ancestry of BV2-5 and bracovirus C-lectin sequences in *Spodotera* spp.

Bracovirus insertions *BV2-5* and *Se-BLL2* were absent from the homologous regions of the *S*. *frugiperda* genomic bacs at NCBI. For *BV2-5* this indicates that the acquisition of bracovirus sequences occurred recently into the *S*. *exigua* genome or more likely that the insertions were probably lost in *S*. *frugiperda*. In accordance with the latter hypothesis a homologous *BV2-5* gene was found to be expressed in *S*. *litura* (Fig 4 and S2 Fig) which is a more recent species in the lineage than *Spodoptera frugiperda* (*S*. *exigua* representing a basal species) [41]. For bracovirus *C-lectins* two clades are present in Spodoptera species suggesting that two events of gene acquisition were both followed by gene expansion. Again the HGT events are probably ancient since sequences of both clades are found in both basal (*S*. *exigua*) and recent (*S*. *litura* and *S*. *litorallis*) Spodoptera species in the lineage, suggesting the lack of *Se-BLL2* sequences in *S*. *frugiperda* is due to gene loss.

**Fig 6:**
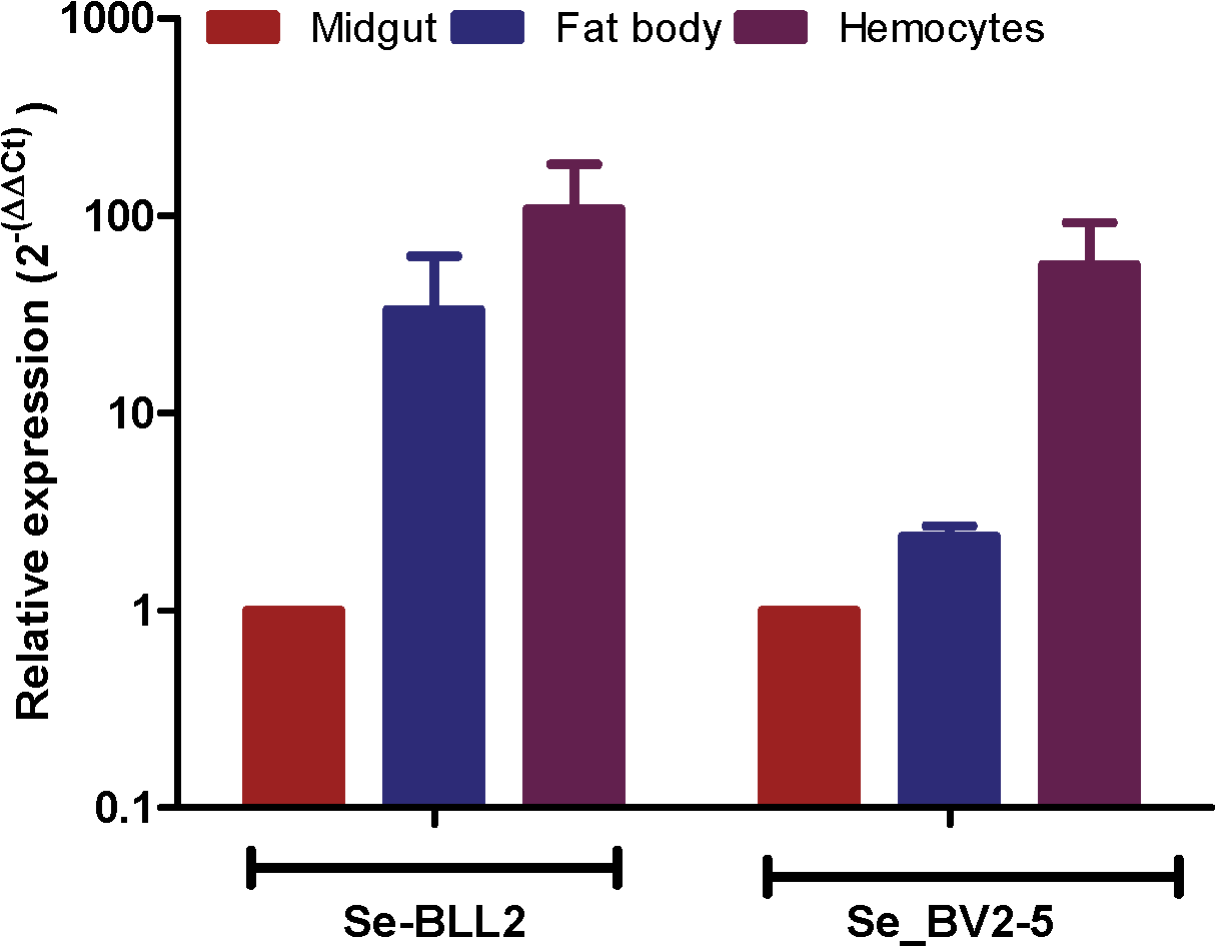
Expression of *BLL2* and *BV2-5* in main larval tissues of *S*. *exigua*. Values were normalized to the *ATP synthase* values and expressed relatively to the abundance in the midgut sample.

The fixation of bracovirus sequences in lepidopteran genomes begs the question of what could be the function of these bracoviral genes in Lepidoptera that could confer a selective advantage. As many bracovirus virulence proteins are interfering with host immunity and as many C-type-lectins are involved in pathogen recognition, we hypothesized that they could modify some features of the lepidopteran immune response resulting in an impact on other pathogens. Indeed, both *S*. *exigua BV2-5* and *BLL2*, although expressed in all the larval tissues tested, are highly expressed in the hemocytes (Fig 6) suggesting that they could be implicated in the immune response of the lepidopteran.

#### BV2-5 interferes with baculovirus motility in the cells and replication

As production of BV2-5 in bacteria was unsuccessful, we used a series of recombinant baculoviruses producing BV2-5 protein or its truncated form (S4 Fig) in an attempt to obtain more information about what could be the role of BV2-5 on the lepidopteran insect physiology.

**Fig 7:**
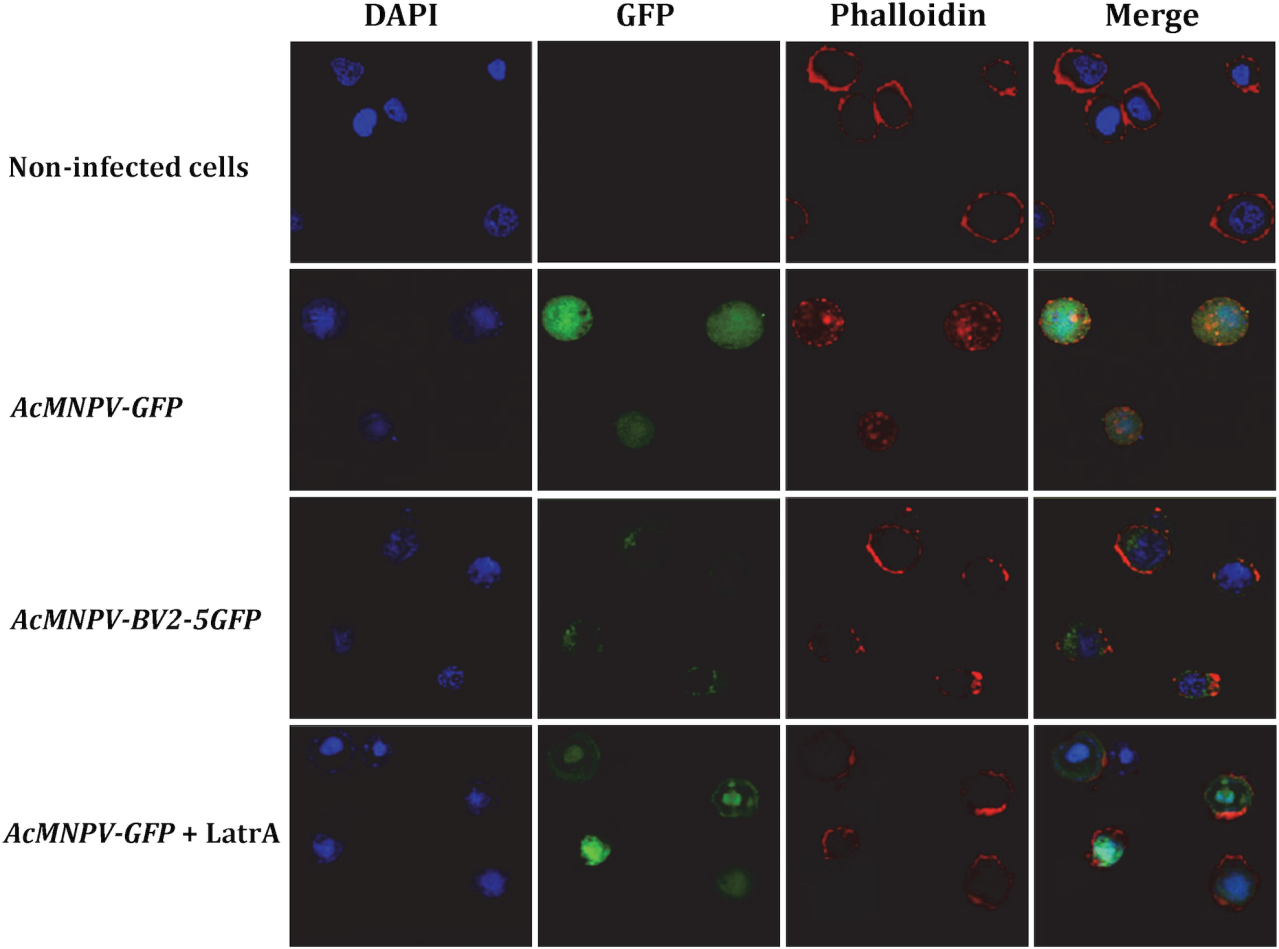
Cellular localization of BV2-5 and its effect on actin distribution. Sf21 cells were infected with different recombinant viruses. The upper horizontal panel represents non-infected cells and the rest represent cells infected with AcMNPV-GFP, AcMNPV-BV2-5GFP and AcMNPV-GFP treated by latrunculin A, respectively. The fluorescence was visualized by confocal microscopy. Nuclei are visualized by DAPI and actin is visualized by phalloidin-TRITC staining.

As a first approach to provide some indication about the role of BV2-5, we studied its cellular localization after infection with baculovirus. Two recombinant viruses were generated: one producing BV2-5 fused to GFP and a control virus expressing GFP (S4A and S5A Figs). Sf21 cells were originally derived from ovarian cell cultures of *S*. *frugiperda* and their genome do not contain BV2-5 gene. These cells were infected with the recombinant viruses and localization of BV2-5 was investigated by following the green fluorescence by confocal microscopy (Fig 7). Confocal observations revealed that BV2-5-GFP was restricted to the periphery of the cells (column GFP, line AcMNPV-BV2-5GFP), while GFP produced from the control virus was homogeneously distributed in all the cellular cytoplasm (column GFP, line AcMNPV-GFP) suggesting BV2-5 has a negative impact on cytoskeleton rearrangement that takes place during baculovirus infection [42]. As previously described during baculovirus infection [42], we observed actin polymerization and changes in its distribution inside the cell, after infection with the control virus and actin staining using phalloidin-TRITC (column Phalloidin, line AcMNPV-GFP). By contrast, actin distribution in Sf21 cells infected with the BV2-5-expressing virus remained similar to that observed in non-infected cells (column Phalloidin, lines AcMNPV-BV2-5GFP and non-infected cells), suggesting that BV2-5 impairs cytoskeleton mediated baculovirus motility. Accordingly we obtained the same localisation of actin at the cell periphery after infection by AcMNPV-GFP and treatment of the cells by latrunculin A, an inhibitor of actin polymerization (column Phalloidin, line AcMNPV-GFP+LatrA) indicating BV2-5 mimics the effects of latrunculin on actin distribution in the cell. It is likely that such a default in cytoskeleton rearrangement either by a direct or indirect interaction of BV2-5 with actin may have a dramatic impact on viral motility and in consequence on the outcome of baculovirus infection since viral manipulation of the actin cytoskeleton both during nucleocapsid transport and after viral gene expression is at the core of successful infection and replication, influencing timing of viral gene transcription, genome processing and packaging [43].

**Fig 8:**
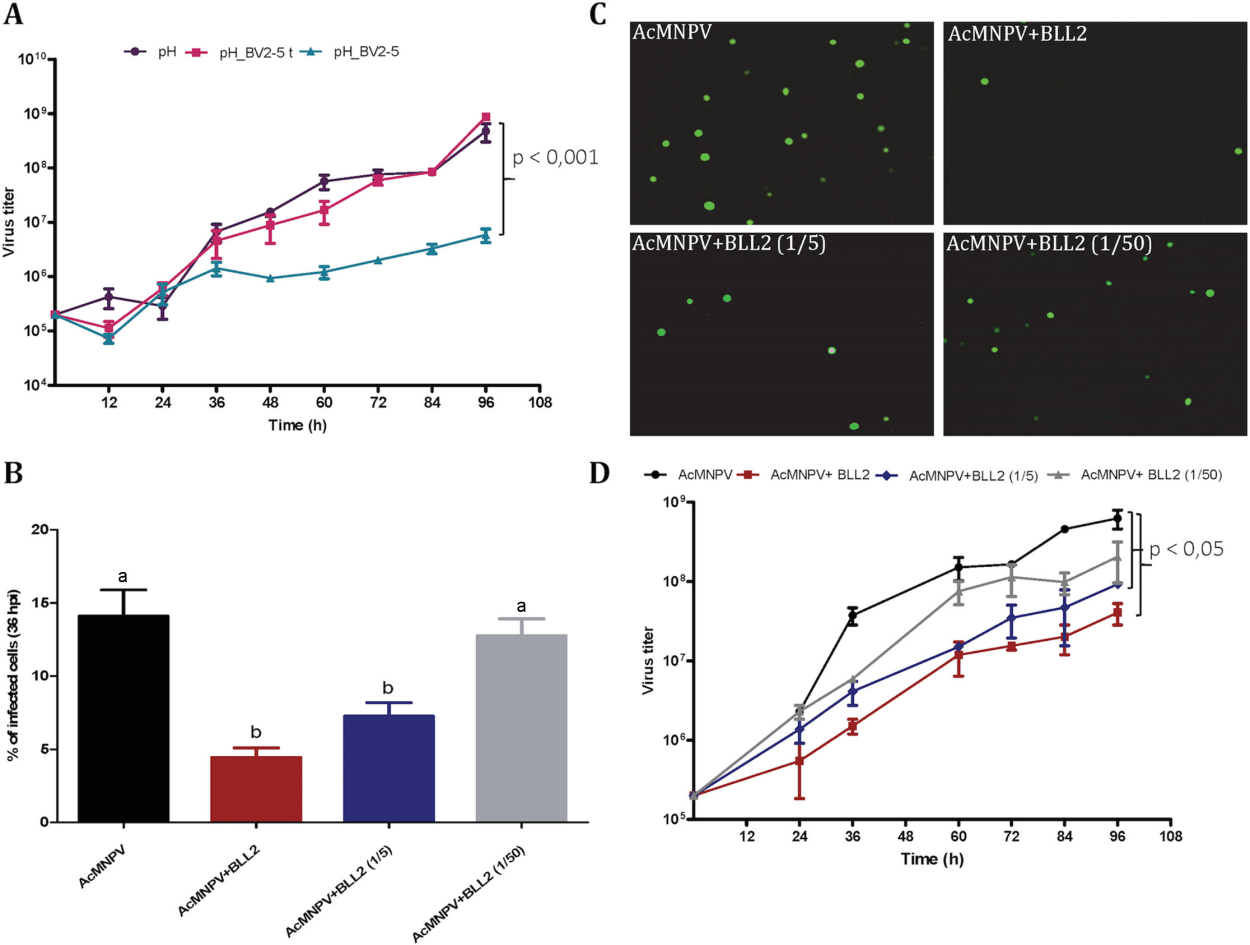
*Spodoptera exigua* bracovirus-like genes affect baculovirus infection. A) Effect of BV2-5 on baculovirus multiplication. One-step growth curve analysis of BV2-5 expressing virus (ph_BV2-5), virus expressing the truncated form (ph_BV2-5 t) and the control virus (ph). The results are the means ± standard deviations (error bars) for independent infection and titration experiments. BV accumulation is shown as the viral titer, calculated for each time point. Statistically different curves and P-Values (Dunnett’s test) are indicated by square brackets. B, C, D) Effect of Se-BLL2 on baculovirus infectivity. AcMNPV-GFP virions were preincubated with different concentrations of purified recombinant Se-BLL2 (50 μg/mL, 10 μg/mL, and 1μg/mL) and then used for the infection of Sf21 cells. B) Percentage of Sf21 cells infected with baculovirus (GFP positive) 36 hours after infection C) Representative images of the infected cells 36 hours after infection. D) One-step growth curve analysis of baculovirus in presence of BLL2. Statistically different curves (Dunnett’s test) are indicated by square brackets.

To assess the hypothesis that BV2-5 expression could impair the multiplication of the virus we compared baculoviruses expressing the BV2-5 protein or its truncated form (BV2-5t) (S4B Fig). The progression of baculovirus multiplication was reduced for the BV2-5-expressing virus compared to the control virus. At 96 hours post infection, the concentration of viral particles allowing cell-to-cell spread of infection (budded virus or BV) in the cell culture medium was reduced by more than two orders of magnitude in the baculovirus producing BV2-5 (Fig 8A). In contrast, the recombinant virus expressing the truncated form (BV2-5t) did not show any significant difference with the control virus. A formal demonstration of the role of BV2-5 would require the production of the protein independently of the baculovirus for which multiplication is tested, however this result together with the effect observed on actin cytoskeleton reorganisation suggest BV2-5 expression in *S*. *exigua* limits baculovirus multiplication. BV2-5 could play a protective role for the Lepidoptera by limiting the impact of baculovirus infection.

If BV2-5 does indeed play a protective role, one might expect that *S*. *exigua* BV2-5 bearing strain (Mexican population) is actually less susceptible than the European population bearing the BV2-5 truncated form. We performed infections using SeMNPV the baculovirus encountered in the field by *S*. *exigua* (S6 Fig) and accordingly significant reduction in SeMNPV virulence was observed in the Mexican population (harbouring BV2-5) when compared to the European population (BV2-5 truncated form). The protection potentially conferred by BV2-5, is however not a complete resistance. Although genetic background between the two lepidopteran strains might be different and several genes might contribute to this phenotype, these results support the contribution of functional BV2-5 forms in reducing susceptibility to baculovirus infection in *S*. *exigua* populations.

#### BLL2 blocks baculovirus infection

Se-BLL2 is probably a secreted protein since the bracovirus homologue C-lectin was shown to be secreted in the haemolymph (cell free) of its Lepidopteran host [44] and the signal peptide for secretion is conserved (residues 1 to 15 according to signalP 3.0 [45]), it could thus interact with a pathogen, a usual function for C-lectins and avoid its dissemination in the infected organism.

Se-BLL2 was expressed in *E*. *coli* and purified using affinity chromatography (S5B Fig). Then we assessed whether Se-BLL2 could have an effect on baculovirus infection using an *ex-vivo* assay allowing good experimental standardization. For that purpose, AcMNPV-GFP virions were preincubated with different concentrations of purified Se-BLL2 and then used to infect Sf21 cells. Baculovirus infectivity was measured as the percentage of GFP positive cells at 36 hpi. Virus preincubation with Se-BLL2 negatively affected viral infectivity in a dose-dependent manner. A reduction of about 65% in viral infectivity was observed for the highest dose of Se-BLL2 (Fig 8B and 8C).

**Fig 9:**
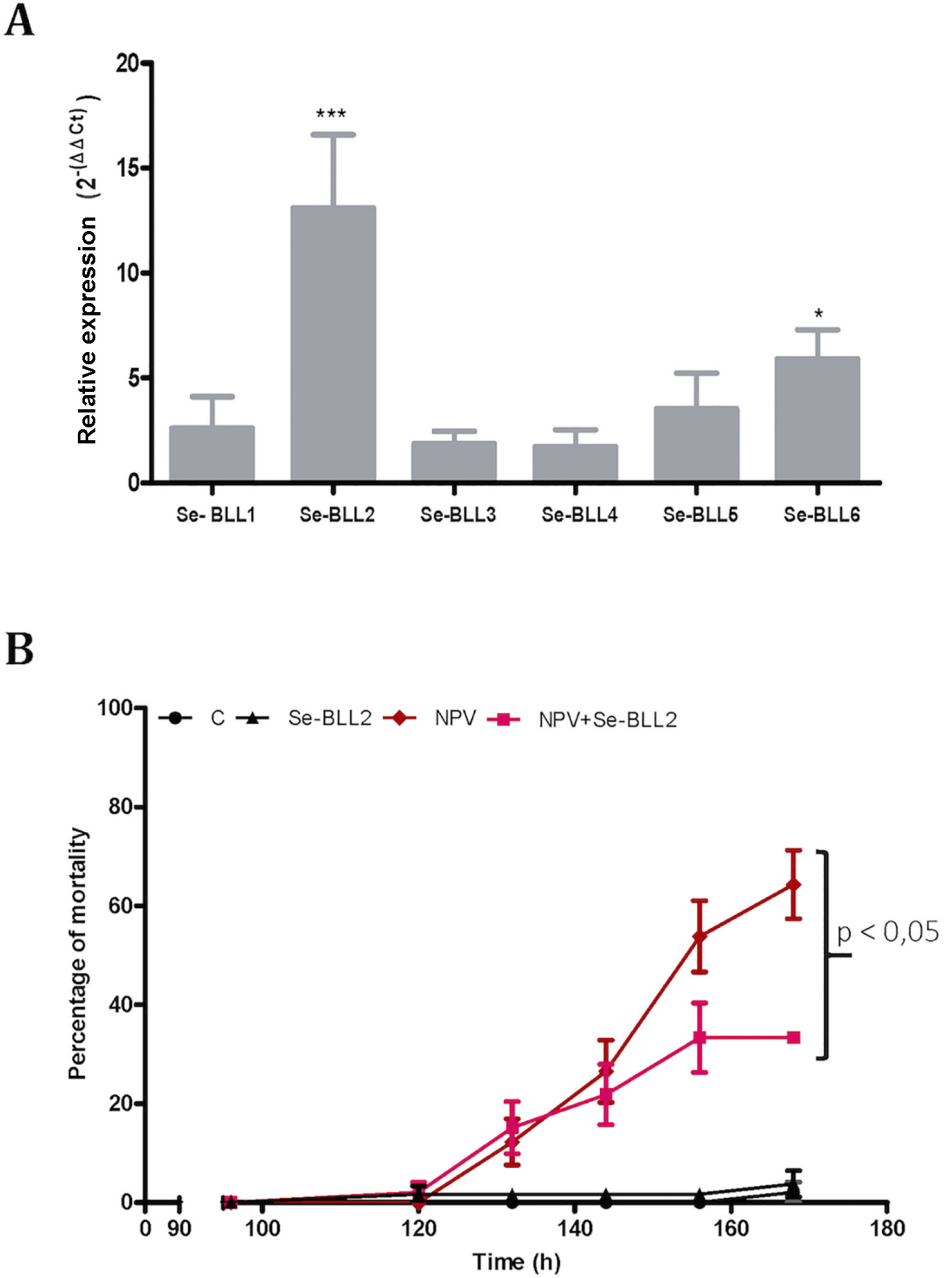
*BLL* expression and protection against baculovirus infection in *S*. *exigua*. A) Changes in the expression of the *BLL* genes after baculovirus infection in the midguts of third-instar larvae of *S*. *exigua* (L3) B) Effect of Se-BLL2 on SeMNPV infection. The time to death was assessed by comparing the mortality curves using the Kaplan Meier method (GraphPad Prism 5). The statistical significance was determined using the log-rank analysis (Mantel-cox test), C refers to control (non-treated) larvae, Se-BLL2 refers to larvae treated with purified Se-BLL2, NPV refers to larvae treated with *S*. *exigua* baculovirus SeMNPV and NPV+Se-BLL2 refers to larvae treated simultaneously with SeMNPV and Se-BLL2 (0. 15mg/mL).

To evaluate whether this effect could also operate *in vivo*, we first tested the effect of infection by the native baculovirus (SeMNPV) on the expression of the *BLLs* in the midgut of *S*. *exigua*, where the primary infection with baculovirus occurs. Compared to non-infected larvae, viral infections induced striking up-regulation of *Se-BLL2* (13-fold) and *Se-BLL6* (5-fold) genes (Fig 9A). These results prompted us to suggest that Se-BLL2 could indeed contribute to protect the larva against the establishment of the baculovirus infection. Then third instar larvae were orally infected with native baculovirus (SeMNPV) in the presence of the recombinant Se-BLL2 protein and larval mortality was registered at different time points. When purified Se-BLL2 was added to the viral inoculum (Fig 9B) larval mortality was reduced by half compared to larvae infected with baculovirus only.

As previously observed for BV2-5, these results suggest that Se-BLL2 expression in *S*. *exigua* might play an important role in conferring some protection or tolerance against baculovirus, a deadly and common lepidopteran pathogen encountered by larvae in the field.

## Discussion

Unexpected levels of similarities were observed between sequences of several lepidopteran genomes and bracoviruses. The level of similarity is in the range of that found for homologous genes coding for highly conserved proteins such as histone H4, almost invariant from plants to animals. However this similarity is unlikely to be due to conservative selection since the encoded genes are conserved only in a limited number of phylogenetically closely related lepidopteran species. In this study we report the presence of these bracovirus-related sequences in several lepidopteran genomes and discuss the possible mechanisms involved in their acquisition. Compared to a previous report describing bracovirus DNA insertions in the monarch and silkworm genomes [31] we provide here an in depth analysis of the structure of the bracoviral and lepidopteran flanking sequences. We show that monarch insertions are fixed in the species, that their presence in the lineage is ancient and that they have undergone rearrangements since their integration. By measuring selection pressures using genomes from individuals of 80 monarch and 8 related species we show that the selection acting on these genes is mainly conservative, which suggests the domesticated *Ben* genes could play a role in monarch physiology. In addition we report for the first time HGT and domestication of bracovirus sequences in Lepidoptera of the *Spodoptera* lineage. Moreover we present functional analysis on 2 unrelated genes suggesting the transferred genes could protect the Lepidoptera against a viral pathogen.

High similarities observed could be due in theory either to DNA sequence transfer from bracovirus to lepidopteran genomes or *vice versa*. Wasp larvae containing a bracovirus as an endogenous virus have an intimate relationship with Lepidoptera since they develop within the body of their hosts, for this reason it is possible that acquisition of lepidopteran genes by bracoviruses can occur. Accordingly horizontal transfer of a Mariner like transposable element (MLE) shared by a parasitoid wasp and its host was previously reported. In this case, the direction of the transfer was supposed to be from Lepidoptera to Hymenoptera based on the presence of this transposon in closely related species of the lepidopteran host and its absence in a closely related parasitoid species [46]. Another horizontal transfer of a transposable element (Helitron) was reported between *Copidosoma floridanum* an endoparasitoid wasp (not associated with a bracovirus) and the Lepidoptera *Trichoplusia ni* suggesting that parasitism might favor horizontal transfer of TEs [47] but the direction of the transfer was not determined in that study. Similarly, Thomas et al., (2010) also found evidence of horizontal transfer of Helitrons in bracoviruses and *Bombyx mori* [48].

One of the insertions described here is particularly informative regarding the direction of the transfer because it contains a regulatory sequence typical of bracoviruses (see Fig 5). The sequences named Direct Repeat Junction (DRJ) that terminate all bracovirus proviral segments are conserved among BVs [18]. These direct repeats are involved in dsDNA circle production [49]. During viral replication, large molecules are amplified that serve as precursors for the production of individual circles, produced by a recombination between the DRJs [19]. As a result, a single DRJ (resulting from the recombination) is present on a circle. This recombination process was confirmed recently by inactivation of two Tyrosine recombinase genes (*vlf1* and *int-1*) using RNA interference, which resulted in impairment of circle formation [50]. The presence of a DRJ in the BV2-5 insertion in the *S*. *exigua* genome constitutes an unambiguous signature of its bracoviral origin since these regulatory elements are specific of the bracovirus life cycle. This clearly demonstrates that the BV2-5 sequence originated from the bracovirus and was acquired by the lepidopteran genome.

The direction of the other horizontal transfers, although not as clearly proven, also appears to be more likely from bracovirus to Lepidoptera genomes, because only a limited number of closely related lepidopteran species harbour these sequences. Moreover bracovirus life cycle features suggest they are involved in horizontal transfer. Indeed bracovirus circles have been shown to enter cells of all tissues tested [51, 52] and to integrate into the DNA of lepidopteran host cells as a part of the wasp life cycle [27]. Several components of the virus particles belong to the integrase family (VLF1, INT1, INT2) [53] and thus potentially mediate integration. During parasitism, bracoviruses do not replicate in host tissues and therefore integration into host cell DNA may allow persistence of bracovirus DNA in lepidopteran larvae that continue to develop [27]. It was previously shown that a side effect of this integration mechanism was to allow circle integration events back into germline cells of the wasp [26]. This was indicated by the analysis of bracovirus sequences in *Cotesia sesamiae* genome. Strikingly, segments homologous to CcBV circle 10 were found in two different genomic locations in *C*. *sesamiae* strains of Kenya [26]. Sequence comparison of circular and reintegrated viral forms [13, 26] indicated that circle integration likely involved the same mechanism as the one described for the integration of bracovirus circles into lepidopteran host genomic DNA during parasitism, using specific sites on the circle (the Host Integration Motifs) [27].

The occurrence of circle integration into lepidopteran host germline DNA resulting in sequence transfer between bracovirus and Lepidoptera is likely another consequence of this viral integration mechanism. Although we did not find integration of complete circles such as those described in the wasp genome, the largest *Ben9* encoding region in *Danaus plexippus* corresponds to more than half of C25 sequence and has retained in this Lepidoptera the bracovirus organisation with two genes (*RnaseT2* and *Ben9*) separated by non-coding sequences [18]. We hypothesize that bracovirus insertions correspond to remnants of circles integrated in Lepidoptera genomes that have been subject to many rearrangements since their integration. Indeed it is likely that after circle integration bracovirus sequences are lost, unless they provide a selective advantage to the insect. Therefore, identification of complete circles in genomes, corresponding to recently integrated sequences, not fixed in the species, might require more diverse template sources than the very limited number of individuals used for lepidopteran genome sequencing. The insertions described in this paper are most probably all ancient. For example, *Ben9* was already present in the common ancestor of the *Danaina* subtribe 5 MYA [35]. Moreover evidence that rearrangements have occurred is provided by the comparison of the two *Ben9* gene containing regions, one having conserved a larger part of the bracovirus non-coding sequence than the other. The insertion in the *B*. *mori* genome has also been obviously rearranged since a stretch of lepidopteran specific DNA separates bracovirus sequences in two parts. BV2-5, Se-BLL2 and SF2.5 insertions in *Spodoptera spp* correspond mostly to single genes, which could represent an ultimate stage of domestication, most of the sequence of the circle having been lost. It is also possible that a broader mechanism than virus-mediated integration, such as DNA repair, which is involved for example in transgenic mice production [54], might have resulted in the insertion of fragments of bracovirus circles in Lepidoptera. However it should be noted that in all cases described in this study the insertions correspond exclusively to sequences from bracovirus circles: we did not find any stretch of wasp sequence flanking or separating bracovirus DNA sequences in the lepidopteran genomes. Thus although integration of wasp DNA could be possible in theory, given that the wasp larvae develop within lepidopteran hosts, we did not search for, nor find evidence of wasp DNA (non-viral) integration in this study.

**Fig 10:**
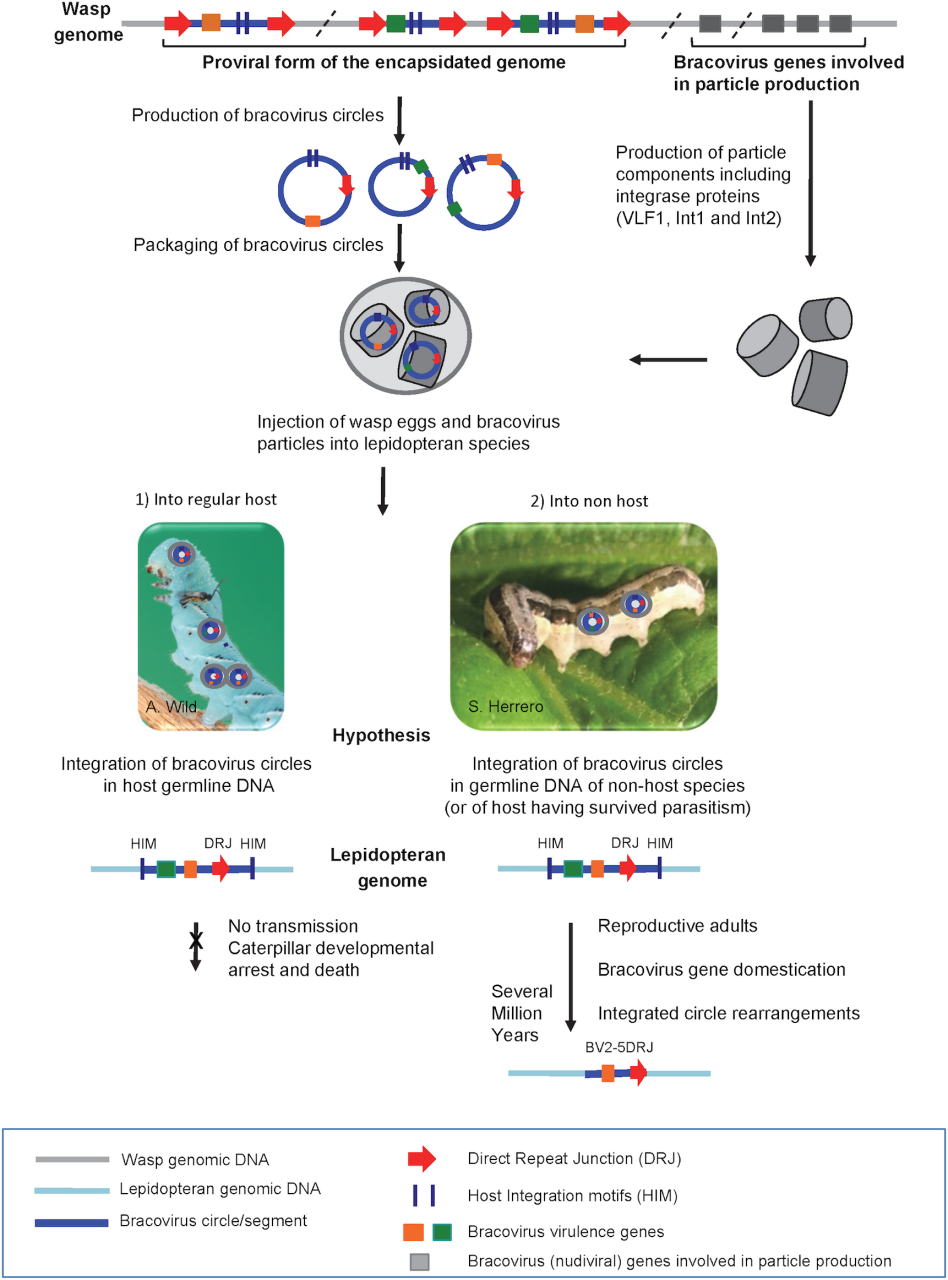
Production of bracovirus particles by the parasitoid wasp *C*. *congregata* and hypothesis on the process leading to transfer of bracovirus sequences to lepidopteran genomes. The BV genome is integrated in the wasp genome (in grey). It is composed of proviral segments (in blue) used to produce dsDNA circles (blue circles) packaged in nucleocapsids (grey cylinders) that encode virulence genes introduced into the host (coloured rectangles) and of BV genes that are involved in particle production (grey rectangles). The latter originate from a nudivirus and encode structural proteins, they are expressed in wasp ovaries where production of bracovirus circles also occurs. Direct Repeat Junctions (DRJ, red triangles) are involved in site-specific recombination allowing circularisation of linear molecules from proviral segments. The circles thus produced are packaged in BV particles that also contain several integrase proteins. The particles are injected in the lepidopteran host during wasp oviposition. Once in the host BV particles infect many lepidopteran cell types but do not replicate. BV circles can integrate into lepidopteran host genomic DNA (in light blue) by a mechanism involving most likely an integrase and mediated by Host Integration Motifs (HIM) indicated by dark blue lines. When injected into a regular host (1) BV virulence gene (coloured squares) expression leads to modifications in lepidopteran host physiology, such as inhibition of wasp egg encapsulation and alteration of developmental programming allowing wasp larvae to complete their development safely in the host body. Hypothesis: when integration of viral circles occurs in the germline the integrated forms are not transmitted because the host dies. When bracoviruses are injected into a caterpillar, which is not a regular host species (2) or is a resistant host (interrupting oviposition, destroying wasp eggs, etc.) the integrated viral form in germline DNA can be transmitted vertically. As bracovirus genes are adapted for expression in lepidopteran cells they can be readily domesticated. Once integrated in lepidopteran genomes the bracovirus sequences undergo rearrangements. Ultimately, after several million years, only the domesticated genes remain from the original integrated circle. We propose that stinging of non-host species could be the main route for bracovirus sequence transfer to Lepidoptera. This is based on the fact that the genome of *M*. *sexta* which is the regular host of *Cotesia congregata* does not contain genes acquired from CcBV, conversely genes found in *Spodoptera exigua*, which is not a host of *Cotesia congregata*, are more closely related to CcBV. This figure is mostly based on the life cycle of CcBV associated with *C*. *congregata* parasitoid wasp of *M*. *sexta*, HIM motifs have been identified in the bracovirus of *M*. *demolitor*, the picture of *S*. *exigua* is shown as an example of *C*. *congregata* non-host species.

The presence of bracovirus sequences in lepidopteran genomes is apparently a paradox given that infected larvae are considered as an evolutionary dead-end (see Fig 10). For example, CcBV has been shown to induce alteration of host developmental programming resulting in inhibition of metamorphosis, even when experimentally injected in a lower amount than during wasp oviposition [28]. Accordingly, we found no evidence for HGT of CcBV genes in *M*. *sexta*, a common host of *C*. *congregata* but instead genes having similarities with other polyDNAviruses [33]. Some host species might be less susceptible to the effect of bracoviruses on development or could have developed resistance mechanisms, and therefore “live to tell the tale” after parasitism and injection of particles (Fig 10). However we propose that the main route of bracovirus gene acquisitions by Lepidoptera could be through parasitoid wasp stinging of non-host species (Fig 10). In the field, the host range of the wasp *C*. *congregata* corresponds to several species of sphingidae, but in laboratory conditions it was shown to oviposit in non-host species such as the noctuidae *Trichoplusia ni* [30]. Such behaviour might offer the opportunity for bracovirus DNA to integrate into genomes of lepidopteran lineages that do not belong to the host range of bracovirus-associated wasps (such as species of the monarch lineage for example) and to “escape” bracovirus induced host development arrest. In this context the cellular machinery of Lepidoptera appear to be sufficiently conserved to express a bracovirus gene normally adapted for expression in a different lepidopteran family. Indeed, the conservation of *Ben9* and *BV2-5* intron splicing in two different lepidopteran families (sphingid/nymphalids and sphingid/noctuids respectively, Fig 1, Fig 2), illustrates that these genes can be “ready to be expressed” even in non-target species. Although not recorded in the field to our knowledge, oviposition in non-hosts may happen since parasitoids, such as *Cotesia* species, attacking aggressive caterpillars do not have the time to intensively examine the potential host before oviposition. In rearing conditions, they sometimes lay their eggs into other adult wasps, which shows the lack of specificity in their choice [55].

Conservation of bracovirus genes in lepidopteran genomes is likely associated with an increase in insect fitness due to the expression of the viral genes. This hypothesis is sustained by functional studies with the SeBLL2 and BV2-5 proteins from *S*. *exigua* showing they have an impact on baculovirus infection. These results suggest that host domestication of these bracoviral genes might increase insect protection to this natural pathogen playing a role in regulating population dynamics in the field [56, 57]. We have found that the interference of recombinant BV2-5 with the cellular cytoskeleton dynamics has a strong impact on the baculovirus producing this protein suggesting BV2-5 could confer larval protection against baculovirus infection. This hypothesis is corroborated by the fact that an *S*. *exigua* BV2-5 bearing strain is less susceptible to baculovirus infection than the European population bearing the BV2-5 truncated form (S6 Fig). However the genetic background between the two strains is probably different and other approaches such as the use of CRISPR/Cas9 technology to produce *S*. *exigua* lines by knocking out of BV2-5 or restoring the functional BV2-5 will be required to unambiguously demonstrate the protective function of this protein, after baculovirus infection. The alteration of a fundamental cellular component such as cytoskeleton dynamics probably also induces a cost. *S*. *exigua* is a Palearctic species, which was introduced in America in 1876 probably from Europe [58]. The fact that Lepidoptera now collected in Europe encode a truncated form of BV2-5 suggests that a recent mutation has spread in this population. It is tempting to speculate that BV2-5-mediated baculovirus protection might induce a cost leading for example to increased susceptibility to other pathogens such as bacteria or parasitoids. The frequency of one or the other form of BV2-5 might depend on the abundance and local selective pressure exerted by pathogens and/or parasites and the cost might also explain why *BV2-5* has been lost in *S*. *frugiperda* while it was detected in *S*. *litura* a more recent species in the *Spodoptera* lineage. In any case BV2-5 coding sequence is more conserved than the other part of the bracovirus insertion suggesting the gene as *Ben* genes in the monarch is generally under conservative selection and not neutrally transmitted.

In addition to BV2-5, we have observed that another gene of bracovirus origin Se-BLL2 can also confer certain level of protection in experimental conditions against both viral forms of baculovirus, occluded derived virions (responsible of the primary infection) and budded viruses (responsible of the systemic infection of larvae). C-type lectins are carbohydrate-binding proteins playing a range of functions in multiple organisms [59]. In general, PDV lectins are able to specifically recognize carbohydrates on the surface of the endoparasitoid eggs and, thus, inhibit the recognition of the eggs by the lepidopteran host recognition proteins [60]. Although little is known about the response of C-type lectins to viral invaders, crustacean lectins have been reported to be related to the antiviral defense [61, 62]. In lepidopterans, the only example of antiviral response involving C-type lectins was reported by Chai *et al*. [63]. Our experiments have shown that BLL2 action is interfering with the initial viral entrance into the Sf21 cells (Fig 8B and 8C). According to these results, it is likely that antiviral action of Se-BLL2 is due to its interaction with viral or host cell membrane glycoproteins involved in viral binding and entrance. Nevertheless, additional studies will be needed to define the exact mode of action of BLL proteins as well as their possible role in the host interaction with viral and non-viral pathogens and parasitoids.

In any case it should be noted that the acquired genes do not confer a complete protection against baculovirus infection and our study confirm that *S*. *exigua* larvae are indeed susceptible to baculovirus infection (Fig 9B). According to the literature the susceptibility of *Spodoptera spp* depend on many factors such as the larval stage [64], the type of plant hosting the insects [65], the geographical origin of the insects, and even on the midgut microbiota composition [66]. Many individuals ingesting a sublethal dose of OBs can survive with a covert infection (larvae harbouring baculovirus but not displaying the disease symptoms) the incidence of which can be over 50% in the field for *Spodoptera exigua* [56]. Little is known on the molecular aspects of this phenomenon but BV2-5 effect on cytoskeleton dynamics could possibly contribute to this latency. Taken together a large number of factors can modulate insect susceptibility and given the high incidence of baculovirus infection in the field being even only less susceptible can have a great impact at the population fitness. In the context of a host-pathogen arms race any new trait that confers an advantage to any of the competitors is susceptible to be incorporated into the gene pool.

Altogether our results strongly suggest that two acquired genes can confer an advantage against viral infection although the comprehensive analysis of the molecular function of the identified proteins is awaited and we cannot completely exclude at this stage that they could have other functions. *Ben* genes also probably have a role for the Lepidoptera since they have been maintained in different lineages and we have shown that in the monarch they are mostly under conservative selection.

We have described in this report several insertions of bracovirus DNA sequences in a series of lepidopteran genomes. In mammals a few examples have been described of integrated retrovirus receptor genes conferring a specific protection against new infections by related viruses using the same cell entry mechanism [67, 68]. Recently, this concept of genes acquired and domesticated by hosts to protect against related virus infections has been shown to operate also for a Bornavirus (negative strand RNA virus) [69]. Virus resistance conferred by expression of viral genes in plants has also been described. Indeed, transgenic plants expressing viral gene constructs can exhibit resistance to infection by the virus [70, 71].

Here, we extend this concept of an organism using pathogen genetic resources as a protection against other pathogens, to insects. Indeed, we show that domestication of different bracovirus genes most likely confers protection to Lepidoptera against baculoviruses, a common pathogen in the field. What is very original compared to previous reported cases is the use of viral sequences as a protection against a distantly related virus. Indeed, most of the viral sequences inserted into host genomes that were hypothesized to confer a protection are effective against closely related viruses. The protection mechanisms are based on the expression of defective proteins of viral origin that are able to interact with those of the pathogenic virus and thus interfere with cell entry [72], replication [73] or interfere by producing small RNAs inducing destruction of virus transcripts having highly similar sequences [74, 75]. Since baculovirus infection of the host could be lethal for the parasitoid [76], it might be speculated that the function of some of the bracoviral genes domesticated by Lepidoptera was already to protect the parasitized larvae against baculovirus infection. This might provide an explanation for both the unusual ability to interfere with distantly related virus infections and the fact that the bracovirus genes have conserved the same structure after their integration into Lepidoptera genomes.

A specific bracovirus circle integration mechanism into lepidopteran host DNA operating during parasitism and resulting occasionally in circle reintegration into wasp genome has been previously characterized [27]. This mechanism is likely involved in HGTs between Hymenoptera and Lepidoptera, although it is also possible that some of the sequences might have been integrated through DNA repair. Once integrated into lepidopteran genomes, bracovirus genes are readily domesticated by Lepidoptera since they are already adapted for expression in lepidopteran tissues during parasitism. Indeed the majority of the CcBV genes expressed during parasitism were shown to possess an insect structure with an arthropod transcription start site, at least one intron and polyadenylation signals [22] and we showed here that the splicing machinery of different Lepidoptera families can produce the same mRNAs from a bracovirus gene containing introns. Altogether the ability of bracoviruses to mediate integration, the fact that bracovirus gene structure is adapted to expression in Lepidoptera and that bracovirus circles have acquired different gene sets depending on the wasp lineage suggest we are only seeing the tip of the iceberg and that numerous cases of integration and domestication of bracovirus sequences will be identified with the exponential rise of genomic data provided by new generation sequencing. Thus this phenomenon is not merely a curiosity but has most likely played an important role in the arms race between Lepidoptera and their pathogens.

## Materials and Methods

### Identification of bracovirus insertions

Sequences sharing high similarity with Bracovirus sequences in lepidopteran genomes were identified using the 35 CcBV circles [18] as queries in megablast analysis (NCBI) against whole genome shotgun contig data banks (wgs at NCBI) restricted to lepidopteran genome sequences. Unlike the bioinformatic study which recently reported numerous short insertions of bracovirus sequences in lepidopteran genomes [31] we focused here on bracovirus-like sequences more than 1 kb long and encoding at least one gene. A blast analysis between proviral integrated circle sequences and the different contigs identified was used to determine the precise location of high scoring pairs (HSP) reported in Fig 1. HSP and annotated sequences were visualised using DNAPlotter [77]. Homologous transcribed sequences were then searched for in the Transcriptome Shotgun Assembly (TSA) database at NCBI, for *Spodoptera* and *Bombyx* sequences (no data was found in TSA for *Danaus plexippus*). It should be noted that although all the identified insertions are very closely related to CcBV since the donor circle can be identified in many cases, this does not necessarily indicate that *C*. *congregata* was the donor species since only a handful of bracovirus packaged genomes have been sequenced to date among those associated with the estimated 1200 species of the *Cotesia* genus [78].

### Insects used for experimental confirmation of the different insertions

Specimen of Danaina subtribe were kindly provided by David Smith. They were collected randomly in the field in the late 1990s, killed in ethyl ethanoate vapour immediately before storage in 95% ethanol [34]. They were stored for ≈6 years at -20°C then for 9 years at room temperature (D. Smith, personal communication). Adult *D*. *plexippus* were collected in Australia (voucher number, vn: 396), *D*. *genutia* in Thailand (vn: 430), *D*. *chrysippus chrysippus* in Oman (vn: 262), *T*. *septentrionis septentrionis* in Malay Peninsula (vn: 216). More recently, *D*. *plexippus plexippus* caterpillars were sampled in August 2012 in Valcartier, Canada (46°56’52”N, 71°29’50”W).

Four colonies of *S*. *exigua*, derived from different geographic locations, were continuously reared on artificial diet at 25± 3°C with 70±5% relative humidity and a photoperiod of 16h light: 8h dark. The FRA strain was supplied by M. Lopez-Ferber, (INRA St.Christol les Alés, France) [79]. The ALM strain was established from successive collections from southern Spain [80]. XEN-R strain was obtained from cotton fields in Pattville, AL. (USA) and was later selected for resistance to *Bacillus thuringiensis* [81, 82]. SUI population was provided by Andermatt Biocontrol AG (Grossdietwil, Switzerland). The DNA from the Mexican population was provided by P. Caballero (Universidad Publica de Navarra). Finally, DNA representing *S*. *exigua* from Japan was obtained from the cell line Se301 originally derived from insects collected in Japan (Hara, et al. 1995).

### DNA extraction and PCR analyses of insertions in *Danaina* subtribe

After grinding frozen samples in liquid nitrogen, DNAs from *D*. *plexippus* larvae were extracted by C. Béliveau using QiaAMP DNA mini kit (Qiagen) and were sent by M. Cusson (Québec, Canada). For specimens of Danaina subtribe, 20 mg of tissues were dried at 37°C to eliminate ethanol, frozen in liquid nitrogen and ground with a pellet and a mortar previously refrigerated at -80°C. DNA was then extracted using the QiAmp DNA Mini Kit (Qiagen) following the supplier’s instructions. To compensate for partial degradation of DNA from old samples, primers were designed for amplification of short fragments. A 35-cycle PCR (94°C for 60 s; 50°C for 60 s; 72°C for 60 s) was performed with 10 pmol of each primer, 0.2 mM dNTP (MP Biochemicals), 1.5 mM MgCl_2_, 0.5 unit Goldstar (Eurogentec) and 20 ng of genomic DNA. PCR products (8 μl) were run on 1.5% agarose gels. The *EF1α* gene was used as a control of DNA sample quality. Successful amplifications of *Ben9* insertions (Fig 2) were obtained: for *D*. *plexippus* using primers Ben9 12F and Ben9 12R (176 bp fragment, S1 Table); for *D*. *chrysippus and D*. *genutia* using primers Ben9 13F and Ben9 13R (173 bp fragment); for and *T*. *septentrionis* using Ben9 14F and Ben9 14R (123 bp fragment); amplifications of *Ben4* insertion (S1 Fig) was obtained using Ben4 2F and Ben4 2R (223 bp fragment). It should be noted that the PCR analyses did not allow us to discriminate between the two *Ben9* insertions (Fig 1(A) and 1(B)).

### RNA extraction and gene amplification from cDNA

Total RNA was isolated from 5^th^ instar *S*. *exigua* larvae using RNAzol reagent (Molecular research centre, INC) as described in the manufacturer’s protocol. One μg of each RNA was DNase treated (Invitrogen) and reverse transcribed into cDNA with oligo (dT) and hexamer primers using Super Script II Reverse Transcriptase from Invitrogen. *D*. *plexippus* RNA was prepared using TRIzol reagent (Invitrogen) and sent by M. Cusson (Québec, Canada), it was further treated by rDNase (NucleoSpin RNA, Macherey-Nagel) to eliminate residual DNA until no PCR amplification of a control gene could be detected from the sample. For *D*. *plexippus*, a total of 1μg of RNA was reverse transcribed into cDNA with oligo (dT) primers using Super Script II reverse transcriptase or Omniscript RT kit (Qiagen). PCR amplifications from cDNA of the different genes were performed using standard protocols and specific primers (S1 Table). For the *BV2-5* alleles, initial sequences were obtained from the transcriptome of *S*. *exigua* larvae exposed to different types of pathogens [40]. Two primers flanking the coding sequence were designed and used to amplify this sequence from cDNAs originating from different populations and cells. RT-PCR-amplified alleles of *BV2-5* were directly sequenced or cloned into pGEM-T Easy vector (Promega) and sequenced using standard primers. At least two independent clones were sequenced for each insect population.

### Measure of the selection pressures operating on *Danaus plexippus Ben4* and *Ben9* genes

Selection pressures operating on *D*. *plexippus Ben4* and *Ben9* genes were measured using Illumina data available at NCBI (SRA data bank) and corresponding to 80 individuals from different wild populations and 8 individuals from other Danaus species (*D*. *erippus*, *D*. *chrysippus*, *D*. *eresimus*, *D*. *gilippus*) [30] in order to identify molecular signatures that might be associated with particular selection pressures. The reads from *D*. *plexippus* samples were mapped onto the reference genome of *D*. *plexippus* [30] with Bowtie2 (2.2.4) [83] using default parameters. The *Ben* regions were then extracted with Samtools [84]. A consensus sequence was obtained for each sample (minimum coverage of 5 reads and minimum frequency of a variant for the individual to be considered as heterozygote = 0.25). Sequences with a missing base ratio above 50% due to heterogeneity in the sequencing were discarded. Reads corresponding to the two *Ben9* copies could not be separated due to high similarity, therefore *Ben9* was analyzed as a single gene. We performed *de novo* assembly of the reads from the other species using Velvet 1.2.07 [85] and a k-mer length of 31 and the *Ben* genes were identified by BLAST. All the sequences were aligned using MAFFT [86], the sequences with a STOP codon were corrected in order to use them for the selection analyses. The global dN/dS (ω) ratios was measured using two different methods, AnalyseCodonData implemented in HyPhy [87] and Codeml from PAML [88] with a means ω ratio for all branches (model = 0) and one ω value for all sites (NSsites = 0). Then, the ω ratio was measured for each site along the alignment, in order to identify regions with a particular selection signature, using the SLAC method implemented in HyPhy (significance thresold: p-value ≤ 0.1) and Codeml from PAML [88] with a means ω ratio for all branches (model = 0) and selection model (NSsites = 2), which classify the sites into three classes (ω = 0 neutral, 0<ω< 1 under negative selection and ω>1 under positive selection). These analyses were performed using the phylogeny based on genome wide SNP data [30]. The phylogenetic tree shown in Fig 3 was built based on the common region of *Ben4* and *Ben9* using PHYML and substitution model HKY85 and 1000 bootstraps.

### Genomic sequence of the *BV2-5* and *Se-BLL2* regions

A universal genome walking kit (Clontech) was used for the sequencing of the whole integrated *BV2-5* and *Se-BLL2* contigs. For this purpose, three *S*. *exigua* genomic DNA libraries [89] were subjected to primary and secondary PCRs using the general primers provided by the kit and specific primers designed to amplify 5’ and 3’ flanking regions of the *BV2-5* and *Se-BLL2* open reading frames (ORFs) (S1 Table). The amplified fragments were purified, cloned into the pGEM-T Easy vector and sequenced.

### Analysis of expression patterns and the response to pathogens

The presence and abundance of mRNA of *Se-BV2-5* and *Se-BLL2* in different larval tissues were analyzed by quantitative reverse transcription PCR (qPCR). Briefly, total RNAs from fat body, midgut and hemocytes were isolated from untreated 5^th^ instar larvae using the RNAzol reagent (Molecular research center, INC) as described in the manufacturer’s protocol. A total of 1 μg RNA was reverse transcribed into cDNA with oligo-(dT) primer using SuperScript II reverse transcriptase (Invitrogen). cDNAs were used to determine the level of transcripts for each gene by qPCR. Reactions were carried out using an ABI Prism 7700 thermocycler from Applied Biosystems. SYBR green Ex Taq master mix (Clontech) was employed in a total volume of 20 μl. Specific primers for each gene were designed by Primer Express Software (Applied Biosystems) (S1 Table). For each gene, at least three biological replicates were employed. Data are presented as fold change using the method of 2^-ΔΔCt^ and normalized to the internal control gene, ATP synthase.

The effect of baculovirus infection on the expression pattern of the lectins in the larval midgut was determined. Third instar larvae were orally infected with *S*. *exigua* nucleopolyhedrovirus (SeMNPV). Each larva was fed with 10^4^ occlusion bodies (OBs). Midguts of treated and untreated larvae were collected 72 h after treatment [66]. Total RNA from the treated and control larvae were collected as described above. cDNA was reverse-transcribed and the presence and abundance of the mRNA was determined using qPCR as described above.

### Generation of recombinant baculovirus

The full ORF of the two main allelic forms of *BV2-5* (complete and truncated) were amplified by PCR from cDNA obtained from *S*. *exigua* FRA and Xen-R larvae, respectively. They were cloned into pFBD-pH vector, downstream of the p10 promoter to generate pFBD-pH-BV2-5 (for the complete form) and pFBD-pH-BV2-5t (for the truncated form) vectors. pFBD-pH refers to the dual vector pFBD (Clontech) containing the AcMNPV polyhedrin gene downstream of the PH promoter. In order to generate recombinant baculoviruses, *Escherichia coli* strain DH10Bac that contains the AcMNPV ΔCC bacmid [90] and the pMON7124 helper plasmid [91] was transformed with pFBD-pH-BV2-5, pFBD-pH-BV2-5t, or pFBD-pH plasmids according to a standard procedure described for the Bac-to-Bac system (Invitrogen). Recombinant bacmids were selected based on white-blue screening of DH10Bac colonies and the positive clones were confirmed by PCR. Bacmid DNAs were isolated from bacterial cells according to standard procedure and used to transfect *S*. *frugiperda* ovary-derived cell line Sf21 using Insect Gene Juice Transfection Reagent (Novagen). Four to six days post transfection, the recombinant ΔCC-pH, ΔCC-pH-BV2-5 and ΔCC-pH-BV2-5t bacmid-derived viruses were collected and multiplied to produce high-titer stocks for further experiments.

Another type of construct was generated to study the cellular localization of BV2-5 by expressing this protein fused to GFP. Primers containing BglII and *Eco*RI were designed to amplify the *BV2-5* gene from the pFBD-pH_BV2-5. The obtained fragment was sub-cloned into pGEM-T Easy, double digested with BglII and EcoRI, and cloned in p166AcV5-Se8-GFP [92] in order to obtain the fusion gene *BV2-5_GFP* in the plasmid p166AcV5-Se8-BV2-5GFP. Subsequently, the *GFP* gene and the recombinant *BV2-5GFP* gene were amplified using specific primers that contained the NotI and PstI restriction sites (Forward *BV2-5GFP*: ^5’^ TTGCGGCCGCATGTTGCCTATTACC^3’^; Forward *GFP*: ^5’^ CTGCGGCCGCATGGGCAAAGGAGAAGAACTTT^3’^; Reverse: ^5’^AGCTGCAGTTACGACCAGCCGCCGCTGGCATCT^3’^). Both genes were cloned under the ph promoter of pFBD to generate pFBD-GFP and pFBD-BV2-5GFP, which were used to transpose into the AcMNPV bacmid as previously described (S4 Fig).

### Cellular localization of the BV2-5 protein by confocal microscopy

Cellular localization of the BV2-5 protein was determined using the recombinant baculovirus expressing BV2-5 fused to GFP (AcMNPV-BV2-5GFP). Previously to the confocal analysis, Sf21 cells were maintained in Grace’s medium (Invitrogen) supplemented with 10% fetal bovine serum and 0.5% penicillin/streptomycin at 27°C. A first set of cells was infected with AcMNPV-BV2-5 GFP and a second set with AcMNPV-GFP at a multiplicity of infection (MOI) of 10. A third set of cells was infected with AcMNPV-GFP and treated with 5 μM latrunculin A (Sigma Aldrich) 12 hours post infection (hpi). A fourth group of cells was maintained without any treatment as a negative control. Seventy-two hpi, cells were pelleted by centrifugation for 2 min at 3000x*g* and fixed with 4% paraformaldehyde (PFA) for 20 min. Then, the cells were washed twice with PBS and permeabilized for 10 min with 0.2% Triton X-100 in PBS-BSA 10%. After another step of PBS washing, cellular actin was stained overnight at 4°C with phalloidin-tetramethylrhodamine B isocyanate TRITC (Sigma Aldrich). Finally, the cells were washed, stained with DAPI (4’, 6’-diamidino-2-phenylindole) to visualize the nucleus of cells and fixed by dakocytomation fluorescent mounting medium (Dakocytomation). Mounted cells were observed under confocal microscope (FV1000, OLYMPUS).

### Effect of *BV2-5* on baculovirus multiplication

Effect of BV2-5 on baculovirus multiplication in cell culture was determined by a one-step growth curve assay. Sf21 cells were infected with the different recombinant baculoviruses at an MOI of 2. After infection, cells were washed and incubated in fresh medium. At different time points, an aliquot of medium was harvested and the viral titer (amount of budded viruses) in each sample was determined by qPCR. For that purpose, viral DNAs were extracted using Prepman reagent (Applied Biosystems) following the manufacturer protocol and were quantified by comparing the obtained Ct values against a standard curve of known viral concentration. Three independent replicates were performed for each sample.

### Insect bioassay and effect of Se-BLL2 on susceptibility to baculovirus

Recombinant Se-BLL2 was expressed and produced in an *Escherichia coli* expression system and purified with affinity chromatography using the HiTrap Chelating HP column (GE Healthcare).

In order to test the effect of BLL2 on baculovirus infection, the purified protein was added in different concentrations (50 μg/mL, 10 μg/mL, and 1μg/mL) to AcMNPV-GFP virions in presence of 10mM CaCl_2_ and the mixture was incubated for 2 hours. After that, the mixture of virus-lectin was then used to infect Sf21 cells (MOI of 0.5). Similarly, other sets of cells were infected with AcMNPV-GFP or AcMNPV-GFP incubated with 10 mM CaCl_2_ as controls. Thirty-six hours post infection, percentage of cells showing GFP was determined for the different treatments in order to compare the virus entry to cells. In addition, an aliquot of the medium was harvested at different time points, and the virus titer was determined for each sample and time point by qPCR as described above.

*S*. *exigua* third instar larvae were infected with Spodoptera exigua multiple nucleopolyhedrovirus (SeMNPV) by the drop-feeding method. Occlusion bodies (OBs) (5x10^5^) from SeMNPV were added to a solution containing sucrose and phenol red colorant (10% and 0.05%, respectively) in presence or absence of purified Se-BLL2 (0. 15mg/mL). The larvae were allowed to drink from the virus and control solution in Petri dishes and then transferred individually to the assay plates. Mortality was then recorded every 12 h until death or pupation of all the larvae. Sixteen larvae were used for each treatment and three independent replicates were performed. Mortality was expressed as the percentage of dead larvae. The time to death was assessed by comparing the mortality curves using the Kaplan Meier method (GraphPad Prism 5). The statistical significance was determined using the log-rank analysis (Mantel-cox test). Insect bioassays comparing susceptibility to SeMNPV in two different populations (SUI and MEX) of *S*.*exigua* were performed as described above at a final dose of 10^3^ OBs/larva.

### Phylogenetic analysis of BV2-5 and lectin sequences

The putative ORFs were determined with the EditSeq program from DNASTAR and the homologs in other insect species were obtained using BLAST comparison at NCBI (http://www.ncbi.nlm.nhi.gov), Silkbase (http://silkworm.genomics.org.cn), *Manduca sexta* genome project (http://agripestbase.org/manduca) and LepidoDB (http://www6.inra.fr/lepidodb). The predicted amino acid sequences were aligned using the ClustalX software [93] and visualized in GenDoc program [94]. Evolutionary distance was calculated for aligned sequences by Maximum-likelihood method and the phylogenetic trees were conducted with the MEGA5 program [95]. Reliability of an inferred tree was determined using bootstrap test (1000 replicates). For a clearer view of the branches, bootstrap values are reported over 100.

### Accession numbers

For the Lectins comparison, the names and accession numbers of proteins compared were as follows. Sf lectin 3–1 (Sf1H08856-3-1) and Sf lectin 5–1 (Sf2H07501-5-1): *Spodoptera frugiperda* proteins obtained from Spodobase (http://bioweb.ensam.inra.fr/spodobase); littoralis_C2971: *S*. *littoralis* lectin-like protein; Sl_lectin: lectin-like protein from *S*. *litura*; Se-BLL1-6: *S*. *exigua* bracovirus-like lectins (KP406769-74). CsMBV CTL CrPDV HP, CvBV L, CpPDV lectin, CrBV lectin, CcV3, CvBV 2L: C-type-lectins from bracoviruses of *Cotesia* species, (AGO14401.1), (BAC55179.1), (AEE09593.1), (AAS10157.1), (AAO74641.1), (CCQ71085.1), (AEE09562.1), Gi-CTLD, Gi-LRP, Gf CTLD2, Gf CTLD3, Gf CTLD4: C-type-lectins from bracoviruses of *Glyptapanteles* species (ABK56997.1), (ABK56993.1), (ACE75074.1), (ACE75072.1), (ACE75071.1). Nv HLPB, Mr LBP, Mr HLBP1, Mr HLPB, Mr_HLBP C-type-lectins from Hymenoptera, *Nasonia vitripennis*, *Megachile rotundata Megachile rotundata*, *Microplitis demolitor* (XP_001599898.2), (XP_003708137.1), (XP_003701025.1), (XP_003706756.1), (XP_003704952.1) (XP_008555202.1), Se-LL1, 2 and 3: *Spodoptera exigua* lepidopteran-like lectins 1, 2 and 3 (KP406775-77), Bm CTL19: *B*. *mori* C-type lectin 19 (NP_001165396.1), Bm CTL21: *B*. *mori* C-type lectin 21 (NP_001037056.1), Ms IML4: *Manduca sexta* immunolectin 4 (AAV41237.2), Ms_IML 4 2: *M*. *sexta* immunolectin 3(AAV41236.1), Ms IML2: *M*. *sexta* immunolectin 2 (AAF91316.3), Ha CTL8: *Helicoverpa armigera* C-type lectin 8 (AFI47453.1), Ha LCT6: *H*. *armigera* C-type lectin 6 (AFI47451.1), Ha CL2: *H*. *armigera* C-type lectin 2 (ACI32834.1), Of_IML: *Ostrinia furnacalis* immunolectin (ABZ81710.1), Lo IML1: *Lonomia oblique* immunolectin 1 (AAV91436.1), Lo L3: *Lonomia oblique* lectin 3 (AAV91450.1), Ap_CTL: *Antheraea pernyi* C-type lectin (AGN70857.1), Mc_CTL: *Mamestra configurata* C-type lectin (AEA76325.1), Pr_CTL: *Pieris rapae* C-type lectin (AEO52696.1), As_CTL: *Anopheles stephensi* C-type lectin galactose binding (ACP43727.1), Ae_CTL: *Aedis aegypti* C-type lectin (ABF18196.1), Md_UP: *Musca domestica* uncharacterized protein LOC101901048 (XP_005189940.1), Dv_GJ: *Drosophila virilis* GJ17272 (XP_002051932.1), Dm BCTL: *Drosophila melanogaster* C-type lectin 27kD, isoform B (NP_001260046.1), De_GG: *Drosophila erecta* GG24353 (XP_001968708.1), Dm CTL: *Drosophila melanogaster* C-type lectin 27kD, isoform A (NP_608858.3), Dy_GE: *Drosophila yakuba* GE14680 (XP_002087961.1), Dw_GK: *Drosophila willistoni* GK23915 (XP_002064562,1), Dmoj GI: *Drosophila mojavensis* GI15343 (XP_002001743,1).

For the BV2-5 comparision, the names and accession numbers are: S.exigua_BV2-5: *S*.*exigua* BV2-5 (KP406767); S.littoralis BV2-5: *S*.*littoralis BV2-5*; S.litura BV2: *S*. *litura* BV2-5 (GBBY01010418.1); CcBV_BV2-5: *C*. *congregata bracovirus* hypothetical protein 3 segment 25 (CCQ71080.1);; GI_HP1 and GI_HP2: *Glyptapanteles indiensis* hypothetical proteins L1_00460 and L1_00290 (ABK57032.1 and ABK57015.1); GF_CHP1 and GF_CHP2: *Glyptapanteles flavicoxis* hypothetical proteins (ACE75094.1 and ACE75115.1).

*S*. *frugiperda* genomic bacs at NCBI, Genbank acc: FP340419.1 and FP340412.1 for *BV2-5* and *Se-BLL2*, respectively.

## Supporting Information

S1 Fig Analysis of BEN4 encoding insertions in the *Danaina* subtribe. The presence of the *Ben4* encoding insertion in genomic DNA of individuals from *Danaus plexippus* and *Tirumala septentrionis septentrionis* (same individuals as in Fig 2) was assessed by PCR amplification using specific primers. C: control PCR (without DNA).
(TIFF)

S2 Fig ClustalX alignment of BV2-5 proteins from different bracovirus and lepidopteran species. SeBV2-5 refers to *S*. *exigua* BV2-5 found in Asian and North American populations, SeBV2-5t refers to the truncated form of *S*. *exigua* BV2-5 found in European populations. CcBV2-5, GIP and GFP proteins are from *Cotesia congregata*, *Glyptapanteles indiensis* and *Glyptapanteles flavicoxis* wasps, respectively. Accession numbers are indicated in materials and methods.
(TIF)

S3 Fig Bracovirus-related lectins. Sequence alignment of the deduced amino acid sequences of bracovirus-lectin like proteins (BLLs) from different *Spodoptera* species and their homologs from Bracovirus (B), hymenopteran (H), lepidopteran (L) and dipteran (D) species employed for the phylogenetic reconstruction. Gene names and accession numbers are reported in Materials and Methods. A) Alignment of the C-type lectin domain (CTLD, cd00037) B) Alignment of the complete amino acid sequences.
(PDF)

S4 Fig Schematic representation of the recombinant baculoviruses (AcMNPV) generated in this work. A) constructs producing fusion protein BV2-5-GFP and GFP B) constructs producing BV2-5 and BV2-5 truncated proteins and control virus.
(TIFF)

S5 Fig Recombinant expression of BV2-5 and BLL2. A) Detection of GFP and GFP fused BV2-5 by Western blot analysis using anti-GFP antibody in Sf21 cells infected with the AcMNPV-GFP (line 2 and 4) and AcMNPV-BV2-5GFP (line 1 and 3) viruses at 72 h p.i.. Soluble (line 1–2) and insoluble (line 3–4) fractions after cellular lysis were tested separately. B) Bacterial expression and purification of BLL2. SDS-PAGE from the bacterial extract (BE) expressing BLL2 (about 20 kDa) and the fractions (F0-F8) obtained after affinity chromatography (6×His column) at increasing concentrations of Imidazole. BE refers to the bacterial extract loaded onto the affinity column.
(TIFF)

S6 Fig Susceptibility to baculovirus in two populations of *S*. *exigua*. Lower susceptibility of an *S*. *exigua* population carrying BV2-5. Virulence of SeMNPV measured as the Median survival time against two populations of *S*. *exigua*. SUI, refers to a European population carrying the truncated form of BV2-5. MEX, refers to an American population carrying the functional BV2-5 form.
(TIF)

S1 Table Primers used in the study. (DOCX)
