# Supplementary figures and images for: Recurrent Domestication by Lepidoptera of Genes from Their Parasites Mediated by Bracoviruses
Source: PLoS Genet. 2015 Sep 17;11(9):e1005470. doi: 10.1371/journal.pgen.1005470 (PMC4574769; doi:10.1371/journal.pgen.1005470)

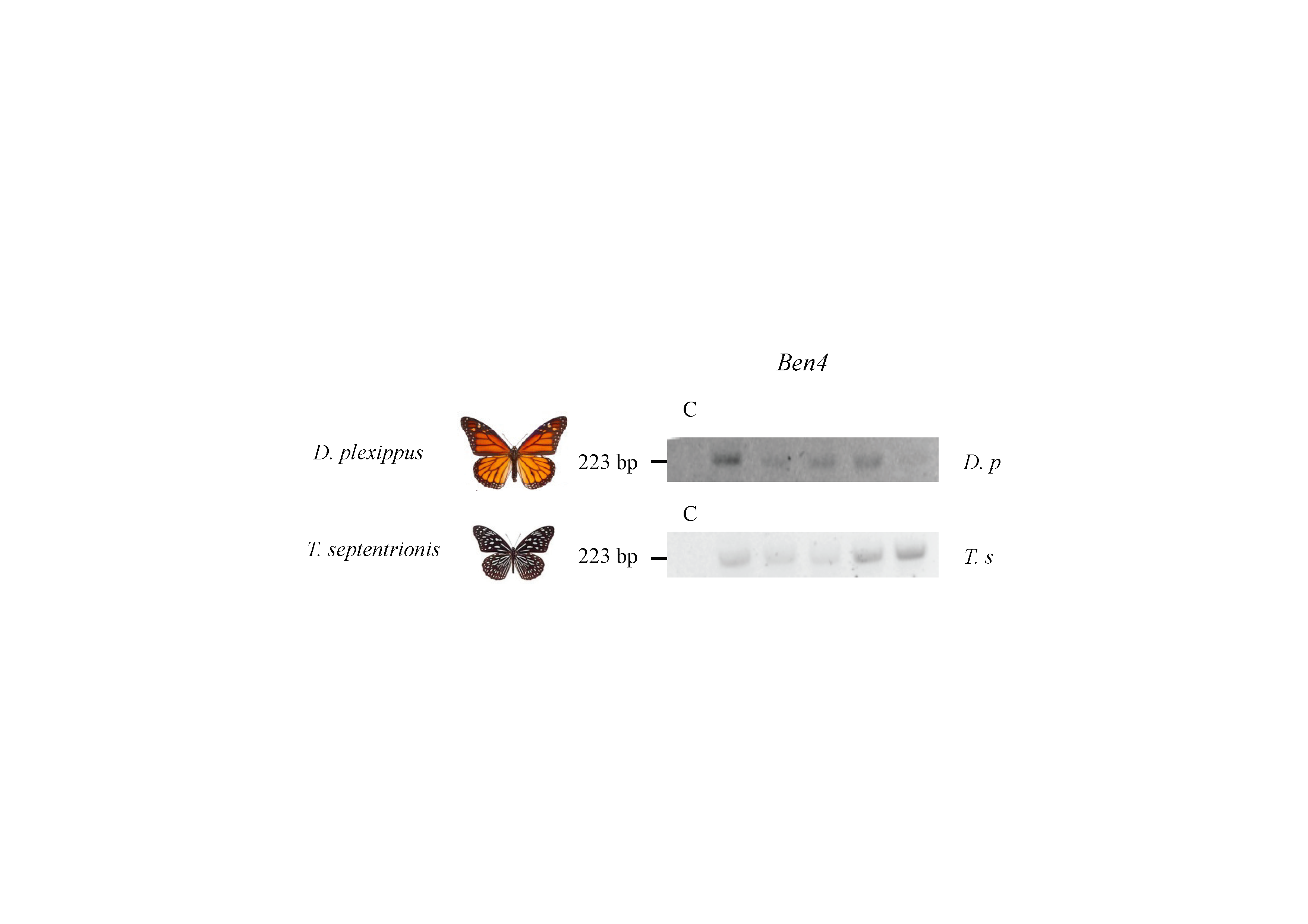

Supplement: S1 Fig — The presence of the Ben4 encoding insertion in genomic DNA of individuals from Danaus plexippus and Tirumala septentrionis septentrionis (same individuals as in Fig 2) was assessed by PCR amplification using specific primers. C: control PCR (without DNA). (TIFF) [file pgen.1005470.s001.tiff]

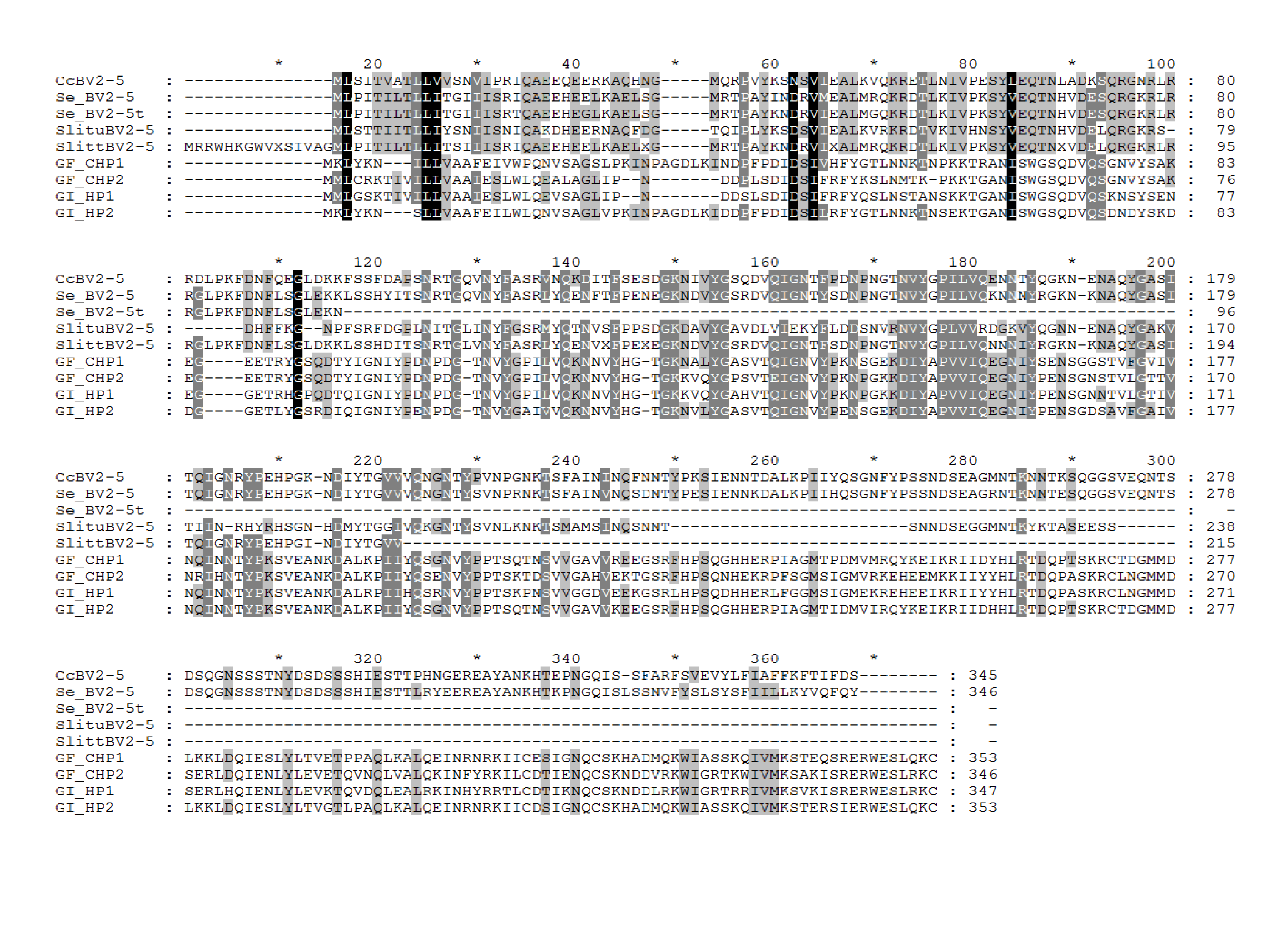

Supplement: S2 Fig — SeBV2-5 refers to S. exigua BV2-5 found in Asian and North American populations, SeBV2-5t refers to the truncated form of S. exigua BV2-5 found in European populations. CcBV2-5, GIP and GFP proteins are from Cotesia congregata, Glyptapanteles indiensis and Glyptapanteles flavicoxis wasps, respectively. Accession numbers are indicated in materials and methods. (TIF) [file pgen.1005470.s002.tif]

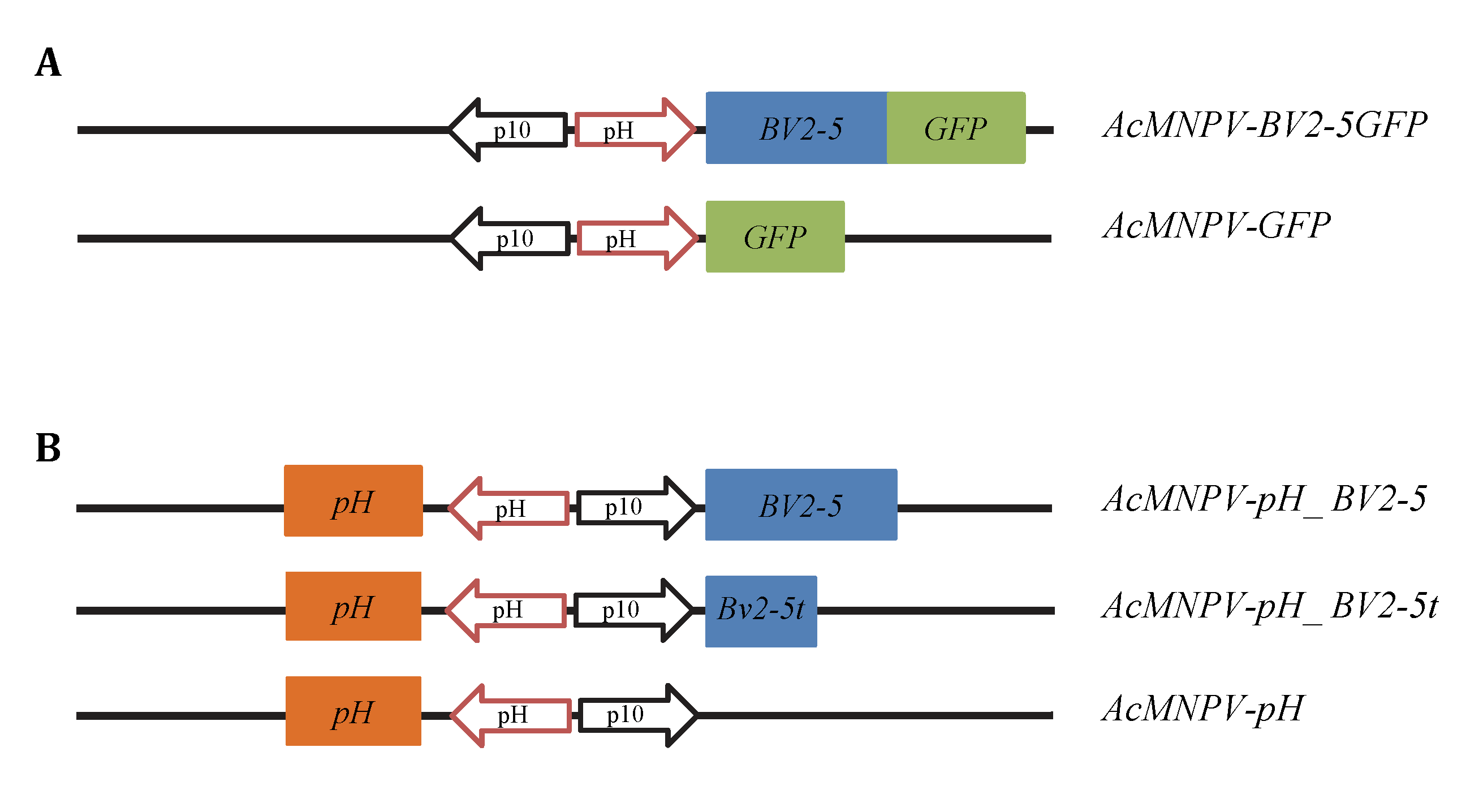

Supplement: S4 Fig — A) constructs producing fusion protein BV2-5-GFP and GFP B) constructs producing BV2-5 and BV2-5 truncated proteins and control virus. (TIFF) [file pgen.1005470.s004.tiff]

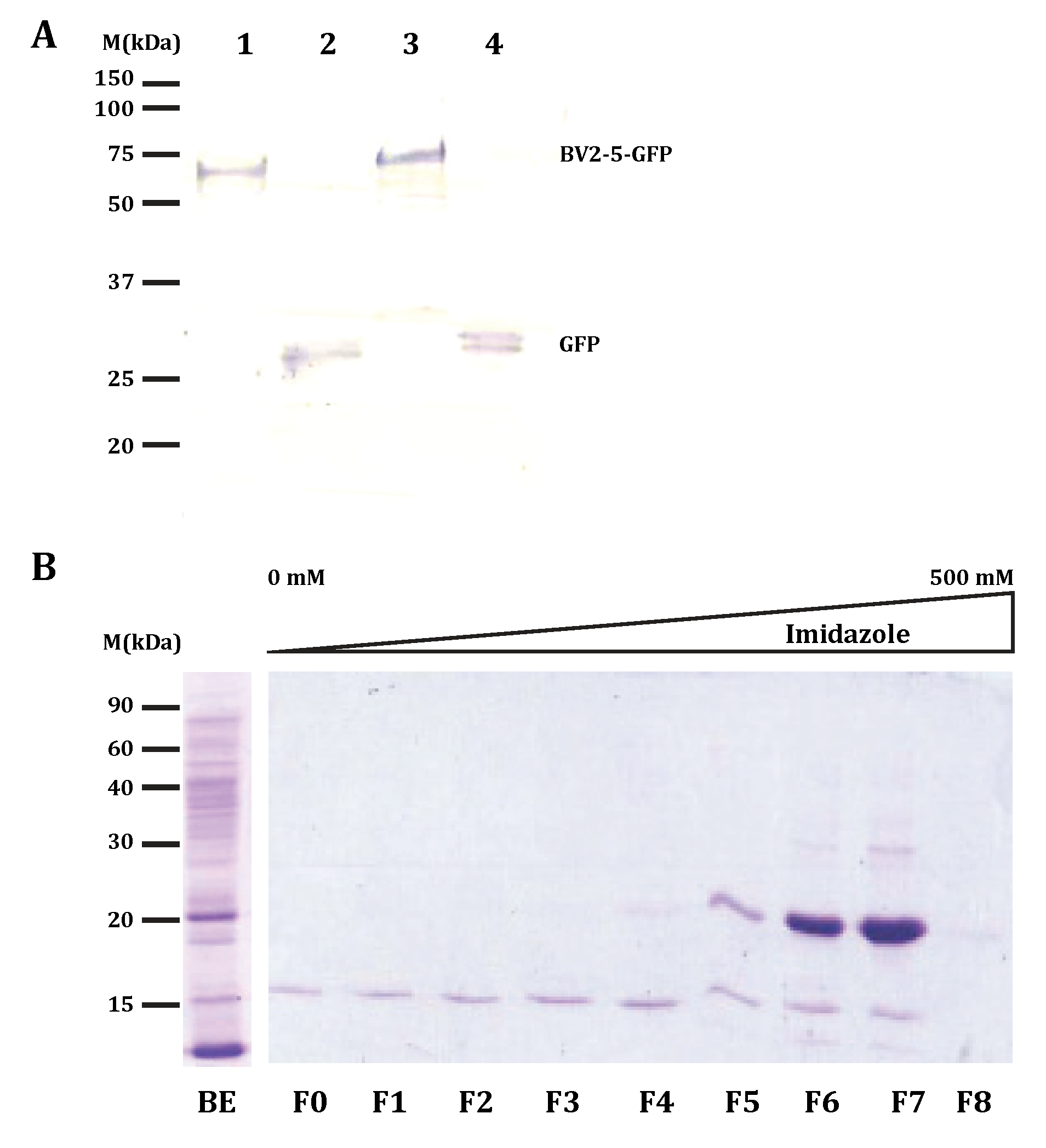

Supplement: S5 Fig — A) Detection of GFP and GFP fused BV2-5 by Western blot analysis using anti-GFP antibody in Sf21 cells infected with the AcMNPV-GFP (line 2 and 4) and AcMNPV-BV2-5GFP (line 1 and 3) viruses at 72 h p.i.. Soluble (line 1–2) and insoluble (line 3–4) fractions after cellular lysis were tested separately. B) Bacterial expression and purification of BLL2. SDS-PAGE from the bacterial extract (BE) expressing BLL2 (about 20 kDa) and the fractions (F0-F8) obtained after affinity chromatography (6×His column) at increasing concentrations of Imidazole. BE refers to the bacterial extract loaded onto the affinity column. (TIFF) [file pgen.1005470.s005.tiff]

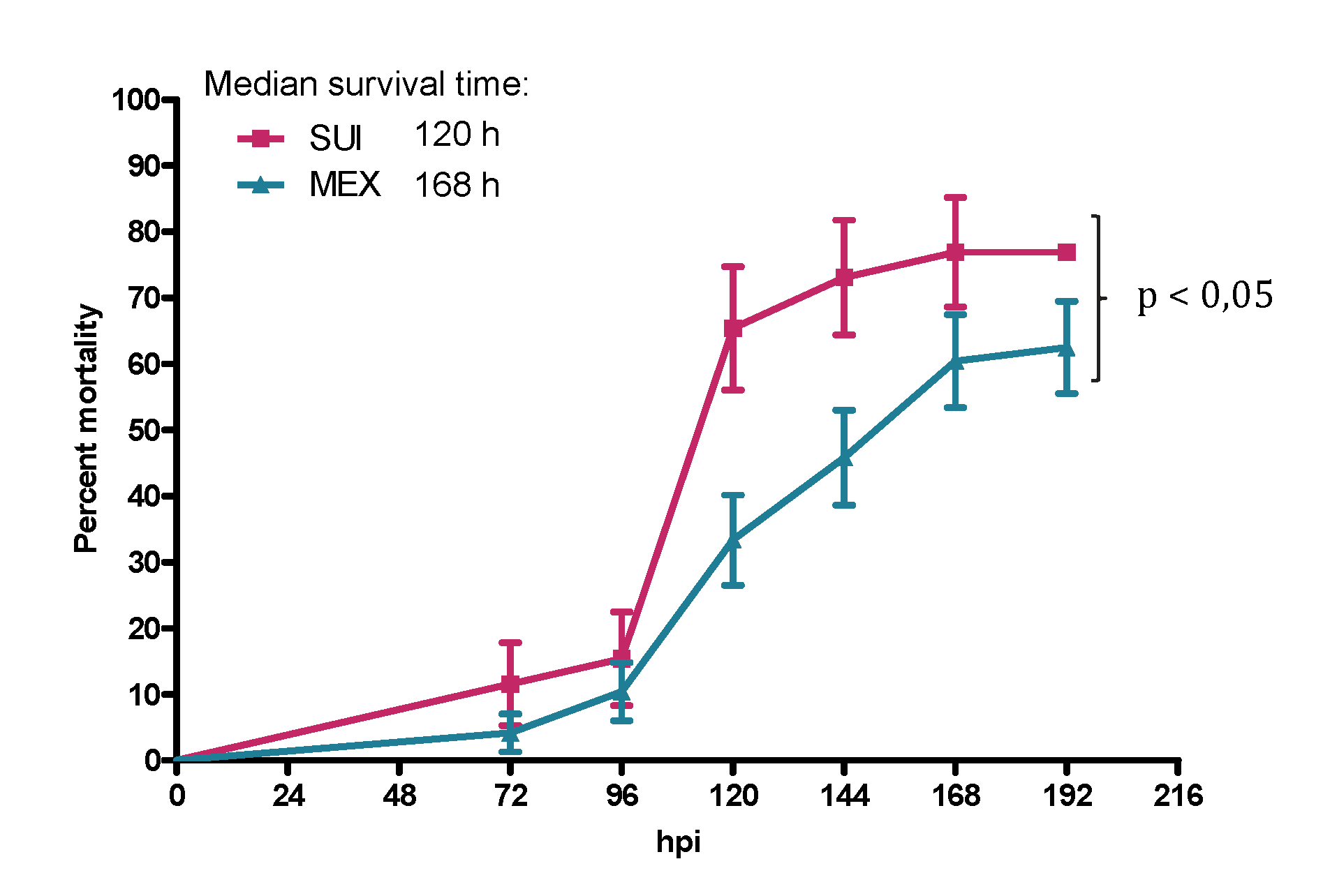

Supplement: S6 Fig — Lower susceptibility of an S. exigua population carrying BV2-5. Virulence of SeMNPV measured as the Median survival time against two populations of S. exigua. SUI, refers to a European population carrying the truncated form of BV2-5. MEX, refers to an American population carrying the functional BV2-5 form. (TIF) [file pgen.1005470.s006.tif]
