# Supplementary material for: Recurrent Domestication by Lepidoptera of Genes from Their Parasites Mediated by Bracoviruses
Source: PLoS Genet. 2015 Sep 17;11(9):e1005470. doi: 10.1371/journal.pgen.1005470 (PMC4574769; doi:10.1371/journal.pgen.1005470)
Supplement: S3 Fig — Sequence alignment of the deduced amino acid sequences of bracovirus-lectin like proteins (BLLs) from different Spodoptera species and their homologs from Bracovirus (B), hymenopteran (H), lepidopteran (L) and dipteran (D) species employed for the phylogenetic reconstruction. Gene names and accession numbers are reported in Materials and Methods. A) Alignment of the C-type lectin domain (CTLD, cd00037) B) Alignment of the complete amino acid sequences. (PDF) [file pgen.1005470.s003.pdf]

A

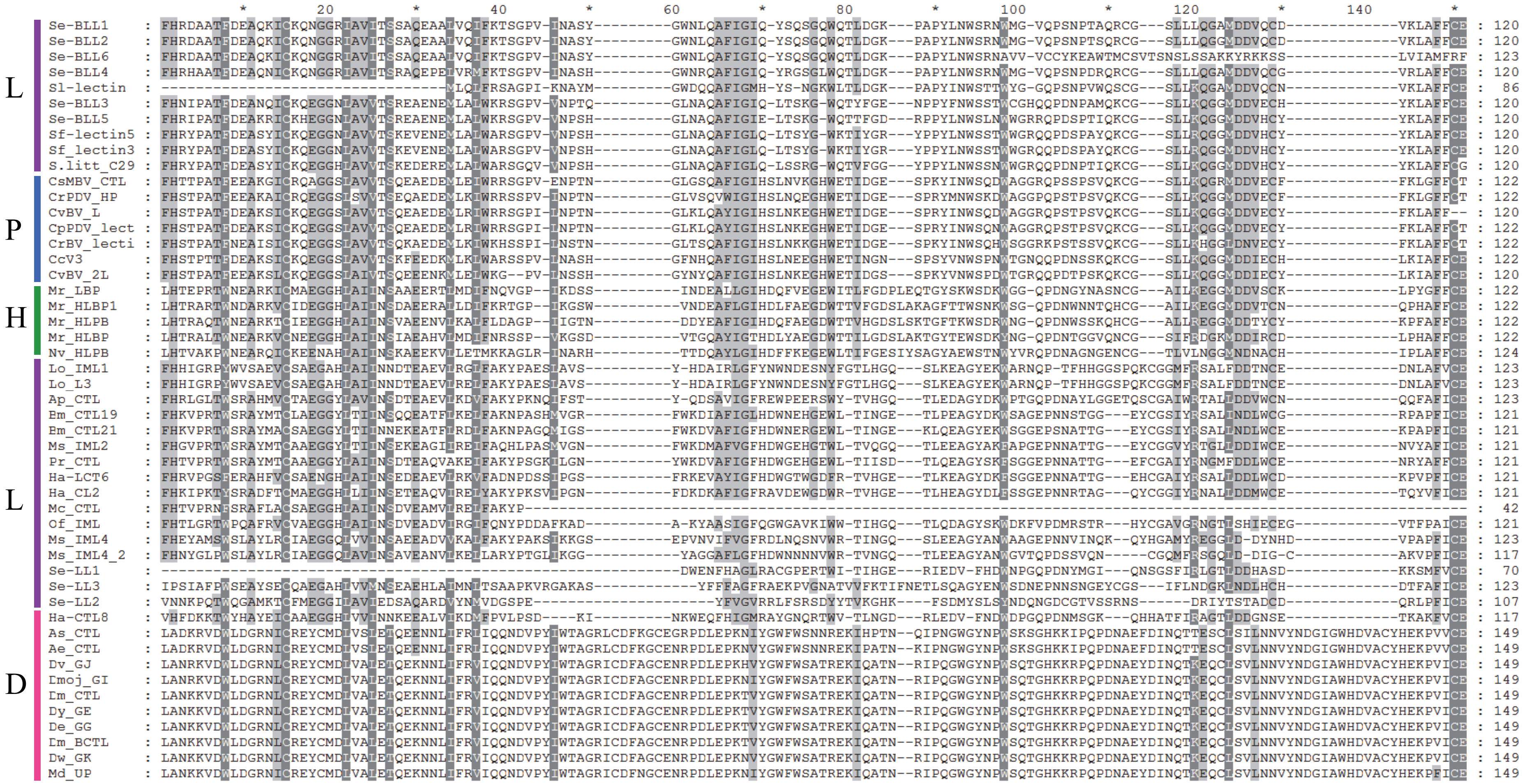
L
P
H
L
D
Se-BLL1
Se-BLL2
Se-BLL6
Se-BLL4
Sl-lectin
Se-BLL3
Se-BLL5
Sf-lectin5
Sf_lectin3
S.litt_C29
CsMBV_CTL
CrPDV_HP
CvBV_L
CpPDV_lect
CrBV_lecti
CcV3
CvBV_2L
Mr_LBP
Mr_HLBP1
Mr_HLPB
Mr_HLBP
Nv_HLPB
Lo_IML1
Lo_L3
Ap_CTL
Bm_CTL19
Bm_CTL21
Ms_IML2
Pr_CTL
Ha-LCT6
Ha_CL2
Mc_CTL
Of_IML
Ms_IML4
Ms_IML4_2
Se-LL1
Se-LL3
Se-LL2
Ha-CTL8
As_CTL
Ae_CTL
Dv_GJ
Imoj_GI
Im_CTL
Dy_GE
De_GG
Im_BCTL
Dw_GK
Md_UP

B

```
                       *        20         *        40         *        60         *        80         *       100         *       120         *       140         *
Se-BLL1     : ----------------------------------------------------------------------------------------------------------------------------------------------------------- :   -
Se-BLL2     : ----------------------------------------------------------------------------------------------------------------------------------------------------------- :   -
Se-BLL3     : ----------------------------------------------------------------------------------------------------------------------------------------------------------- :   -
Se-BLL4     : ----------------------------------------------------------------------------------------------------------------------------------------------------------- :   -
Sf-lectin5  : ----------------------------------------------------------------------------------------------------------------------------------------------------------- :   -
Se-BLL6     : ----------------------------------------------------------------------------------------------------------------------------------------------------------- :   -
Sl-lectin   : ----------------------------------------------------------------------------------------------------------------------------------------------------------- :   -
Se-BLL5     : ----------------------------------------------------------------------------------------------------------------------------------------------------------- :   -
Sf_lectin3  : ----------------------------------------------------------------------------------------------------------------------------------------------------------- :   -
Slittorali  : ----------------------------------------------------------------------------------------------------------------------------------------------------------- :   -
CsMBV_CTL   : ----------------------------------------------------------------------------------------------------------------------------------------------------------- :   -
CrPDV_HP    : ----------------------------------------------------------------------------------------------------------------------------------------------------------- :   -
CvBV_Lecti  : ----------------------------------------------------------------------------------------------------------------------------------------------------------- :   -
CpPDV_lect  : ----------------------------------------------------------------------------------------------------------------------------------------------------------- :   -
CrBV_lecti  : ----------------------------------------------------------------------------------------------------------------------------------------------------------- :   -
CcV3        : ----------------------------------------------------------------------------------------------------------------------------------------------------------- :   -
CvBV_2L     : ----------------------------------------------------------------------------------------------------------------------------------------------------------- :   -
Gf3CTLD     : ----------------------------------------------------------------------------------------------------------------------------------------------------------- :   -
GF-4CTLD    : ----------------------------------------------------------------------------------------------------------------------------------------------------------- :   -
Gf-2CTLD    : ----------------------------------------------------------------------------------------------------------------------------------------------------------- :   -
Gi-CTLD     : ----------------------------------------------------------------------------------------------------------------------------------------------------------- :   -
Gi-LRP      : ----------------------------------------------------------------------------------------------------------------------------------------------------------- :   -
MdHLBP      : ----------------------------------------------------------------------------------------------------------------------------------------------------------- :   -
Mr-HLBP1    : ----------------------------------------------------------------------------------------------------------------------------------------------------------- :   -
Mr-HLPB     : ----------------------------------------------------------------------------------------------------------------------------------------------------------- :   -
Mr-HLBP     : ----------------------------------------------------------------------------------------------------------------------------------------------------------- :   -
Mr-LBP      : ----------------------------------------------------------------------------------------------------------------------------------------------------------- :   -
Nv_HLPB     : ----------------------------------------------------------------------------------------------------------------------------------------------------------- :   -
Se-LL3      : ------------------------------------------------------------------------MKNYVLLVVCLIFSSSEAPPPQVSKQYRTDYEYNRKTDAFYKLHIETT--PHYLVEQLCKVEGAELMVPRSD----------- :  70
Bm_CTL19    : ---------------------------------------------------------------------MKAANKSLVFVLI-------LICSVVGQQFRYDYTYFRNINGWLKLQEIPA--IWQEARLRCRLEGSVLASPLDA----------- :  66
Bm-CTL21    : ---------------------------------------------------------------------MKAALASLVFVLT--------IAYLDGQQFRYDYTYMRDINGWLKLQEIPA--TWQEARLRCHLEGSLLASPLDD----------- :  65
Pr_CTL      : --------------------------------------------------------------------MYR-CIVLTVLFA--------VIASIDARRFRCDYKYFPKAGGYLKLHRIPA--NWREARLRCHLEGTKIASLLNE----------- :  65
Lo_IML1     : MKELATNKKNGVYTGIPGTVSKGDFHSIDGIPISEISLQWLAGDPNNAGNNEYCIIYHANGQAADVDCSRPFPFICYKKHSK-------DMRITECGTIDTEYKLDKRTNKCYKFHHIGR--PYWVSAEVCSAEGAHLAIINNDTEAEVLRGLFA : 146
Se-LL2      : -------------------------------------------------------------------------MKSILILISLLGITLPSAHSRHVPFYRKDYTYIEHFDAFYKVHWDISGTNWNLAFLTCEDEGALLFYPKAQG---------- :  72
Msexta_IML  : --------------------------------------------------------------------MYKSFIFICVYFTS-------SIVSTNHVNFRCDYKYLDVIDGWMKLHEIPA--NWHEARLRCHLEGAVLASPLNS----------- :  67
Lo_L3       : -------------------------------------------------------------------MLAPLTLLFILITAE-------TLCAAEDNQFRADYKYYKEADGWFKVHHMPR--KWQHARLRCAYEGAMLASP-------------- :  65
Ha-LCT6     : ---------------------------------------------------------------------MKTFIKSFVFLLF-------FIYTECA--FRCDYQYRQLTKGWFKLYEVPE--TWQDARLRCTLQGAVLASPTSS----------- :  64
Ha_CL2      : ---------------------------------------------------------------------MENFIKCFVFTFC-------FISIECA--FRCDYKYNIMAKGWFKLYEVPE--TWYDARQRCSLQGAILASPTTP----------- :  64
Of_IML      : --------------------------------------------------------------------MKVSIILVLVFIFYPHHGDSSELNTSKSNWFRPDYSYSERTGGWFKFHSVPA--TWEDARLQCYYEGAELVSPFNEN---------- :  75
Ms_IML4     : ---------------------------------------------------------------------MDAQQIFFVLFAS--------VVVHGSYDFRPDYEYHASAGGWFKLHLVPA--TWSDARFICDFEGAVLASPINV----------- :  65
Ms_IML3     : ---------------------------------------------------------------------MEVLRGVVVLITV--------SIVQGSNVFRADYEYHASAGGWFKFHKVPA--DWHDARLMCDFEGAVLASPINV----------- :  65
Ha-CTL8     : ---------------------------------------------------------------------------------------------TDGVLSKPQYLFSYEAEGWLKLHKFPA--TWEQAFLRCHYEGAVLASPLDLR---------- :  50
Se-LL1      : -------------------------------------------------------------------------MTLY--------------ELSESARCDPHYKFNMDANGWLKVHQIPA--TWEEAFLRCHYEGAVLASPLTQQ---------- :  56
Ap_CTL      : ----------------------------------------------------------------------MYKTFFCVLASS-------ALAFT----FREDYKHYEEANGWIKLHKVPA--NWQDARLRCRAEGASLASPSNS----------- :  61
As_CTL      : ----------------------------------------------------------------------------------------------------------------------------------------------------------- :   -
Ae_CTL      : ----------------------------------------------------------------------------------------------------------------------------------------------------------- :   -
Mc_CTL      : ---------------------------------------------------------------------MKSCFKYFVLLFG-------LNLTKGS--FRCDYKYSSKAQTWFKPFVVPA--NWFDARLRCSMEGAVLASPTST----------- :  64
Md_UP       : ----------------------------------------------------------------------------------------------------------------------------------------------------------- :   -
Dv_GJ       : ----------------------------------------------------------------------------------------------------------------------------------------------------------- :   -
Em_CTL      : ----------------------------------------------------------------------------------------------------------------------------------------------------------- :   -
Em_BCTL     : ----------------------------------------------------------------------------------------------------------------------------------------------------------- :   -
De_GG       : ----------------------------------------------------------------------------------------------------------------------------------------------------------- :   -
Em_GI       : ----------------------------------------------------------------------------------------------------------------------------------------------------------- :   -
Dy_GE       : ----------------------------------------------------------------------------------------------------------------------------------------------------------- :   -
Dw_GK       : ----------------------------------------------------------------------------------------------------------------------------------------------------------- :   -
```

B (continuation)

```
                    160         *       180         *       200         *       220         *       240         *       260         *       280         *       300         *
Se-BLL1      : ----------------------------------------------------------------------------------------------------------------------------------------------------------- :
Se-BLL2      : ----------------------------------------------------------------------------------------------------------------------------------------------------------- :
Se-BLL3      : ----------------------------------------------------------------------------------------------------------------------------------------------------------- :
Se-BLL4      : ----------------------------------------------------------------------------------------------------------------------------------------------------------- :
Sf-lectin5   : ----------------------------------------------------------------------------------------------------------------------------------------------------------- :
Se-BLL6      : ----------------------------------------------------------------------------------------------------------------------------------------------------------- :
Sl-lectin    : ----------------------------------------------------------------------------------------------------------------------------------------------------------- :
Se-BLL5      : ----------------------------------------------------------------------------------------------------------------------------------------------------------- :
Sf_lectin3   : ----------------------------------------------------------------------------------------------------------------------------------------------------------- :
Slittorali   : ----------------------------------------------------------------------------------------------------------------------------------------------------------- :
CsMBV_CTL    : ----------------------------------------------------------------------------------------------------------------------------------------------------------- :
CrPDV_HP     : ----------------------------------------------------------------------------------------------------------------------------------------------------------- :
CvBV_Lecti   : ----------------------------------------------------------------------------------------------------------------------------------------------------------- :
CpPDV_lect   : ----------------------------------------------------------------------------------------------------------------------------------------------------------- :
CrBV_lecti   : ----------------------------------------------------------------------------------------------------------------------------------------------------------- :
CcV3         : ----------------------------------------------------------------------------------------------------------------------------------------------------------- :
CvBV_2L      : ----------------------------------------------------------------------------------------------------------------------------------------------------------- :
Gf3CTLD      : ----------------------------------------------------------------------------------------------------------------------------------------------------------- :
GF-4CTLD     : ----------------------------------------------------------------------------------------------------------------------------------------------------------- :
Gf-2CTLD     : ----------------------------------------------------------------------------------------------------------------------------------------------------------- :
Gi-CTLD      : ----------------------------------------------------------------------------------------------------------------------------------------------------------- :
Gi-LRP       : ----------------------------------------------------------------------------------------------------------------------------------------------------------- :
MdHLBP       : -----------------------------------------------------------------------------------------------------------------------------------------------MFKLIYLILLPI :   1
Mr-HLBP1     : ----------------------------------------------------------------------------------------------------------------------------------------------------------- :
Mr-HLPB      : ----------------------------------------------------------------------------------------------------------------------------------------------------------- :
Mr-HLBP      : -------------------------------------------------------------------------------------------------------------------------------------------------MSLLSSLVNF :   1
Mr-LBP       : ----------------------------------------------------------------------------------------------------------------------------------------------------------- :
Nv_HLPB      : -----------------------------------------------------------------------------------------------------------------------------------------------MIQPLVVSIFAI :   1
Se-LL3       : ---------------------------------------------------------------------------------------------------------------------------------------YDIEQMHGMFKKFPDIGNYV :   9
Em_CTL19     : ------------------------------------------------------------------------------------------------------------------------------------------ALKSSMLSLITNK--KS :   8
Em-CTL21     : ------------------------------------------------------------------------------------------------------------------------------------------ALKSGMLSLIKNK--KT :   8
Pr_CTL       : ------------------------------------------------------------------------------------------------------------------------------------------ELKTAMGSMMDVTP-KL :   8
Lo_IML1      : KYPAESLAVSYHDAIRLGFYNWNDESNYFGTLHGQSLKEAGYEKWARNQPTFHHGGSPQKCGGMFRSALFDDTNCEDTLAFVCEKDAESLVSFNLDNQFGADLTSLRKLMVFLIASHIANGSIKASLLGMAYGRQCSFSTNYGLATVMKELATNK :  30
Se-LL2       : ------------------------------------------------------------------------------------------------------------------------------------------EWALVKNLTEMMTEVPN :   8
Msexta_IML   : ------------------------------------------------------------------------------------------------------------------------------------------NLKFAMASMMILKT-PK :   8
Lo_L3        : -------------------------------------------------------------------------------------------------------------------------------------------TNYGLATVMKELATNK :   8
Ha-LCT6      : ------------------------------------------------------------------------------------------------------------------------------------------AMAAEMRHTMKNFF-LQ :   8
Ha_CL2       : ------------------------------------------------------------------------------------------------------------------------------------------DIEFAMKQTMRNLF-LQ :   8
Of_IML       : -----------------------------------------------------------------------------------------------------------------------------------------------IIQKMIMLMDVN :   8
Ms_IML4      : ------------------------------------------------------------------------------------------------------------------------------------------AVNNVMQSIVNMSDHLS :   8
Ms_IML3      : ------------------------------------------------------------------------------------------------------------------------------------------DVTDVLQNIINKIEHLS :   8
Ha-CTL8      : -------------------------------------------------------------------------------------------------------------------------------------------LVHALRSTMAQNG-VH :   6
Se-LL1       : -------------------------------------------------------------------------------------------------------------------------------------------LSKAIENKFSDIS-CY :   7
Ap_CTL       : ------------------------------------------------------------------------------------------------------------------------------------------NIRNVMISVMIAESVDP :   7
As_CTL       : ----------------------------------------------------------------------------------------------------------------------------------------------LVVASLVAVCLAE :   1
Ae_CTL       : -----------------------------------------------------------------------------------------------------------------------------------------MKLLVLVLASLIAVCLAE :   1
Mc_CTL       : ------------------------------------------------------------------------------------------------------------------------------------------EILAEMR-TLMNRS-YP :   7
Md_UP        : ------------------------------------------------------------------------------------------------------------------------------------------MKVTLVLAAFCVAASYA :   1
Dv_GJ        : ------------------------------------------------------------------------------------------------------------------------------------------MKVTLALATFCLVAACA :   1
Im_CTL       : ------------------------------------------------------------------------------------------------------------------------------------------MKVTLAIATFCVVMACS :   1
Im_BCTL      : ------------------------------------------------------------------------------------------------------------------------------------------MKVTLAIATFCVVMACS :   1
De_GG        : ------------------------------------------------------------------------------------------------------------------------------------------MKVTLAIATFCVVMACS :   1
Im_GI        : ------------------------------------------------------------------------------------------------------------------------------------------MKVTLALATFCLVAACA :   1
Dy_GE        : ------------------------------------------------------------------------------------------------------------------------------------------MKVTLAIATFCVVMACS :   1
Dw_GK        : ----------------------------------------------------------------------------------------------------------------------------------------MRMKVTLAIATFCVMAAYS :   1
```

B (continuation)

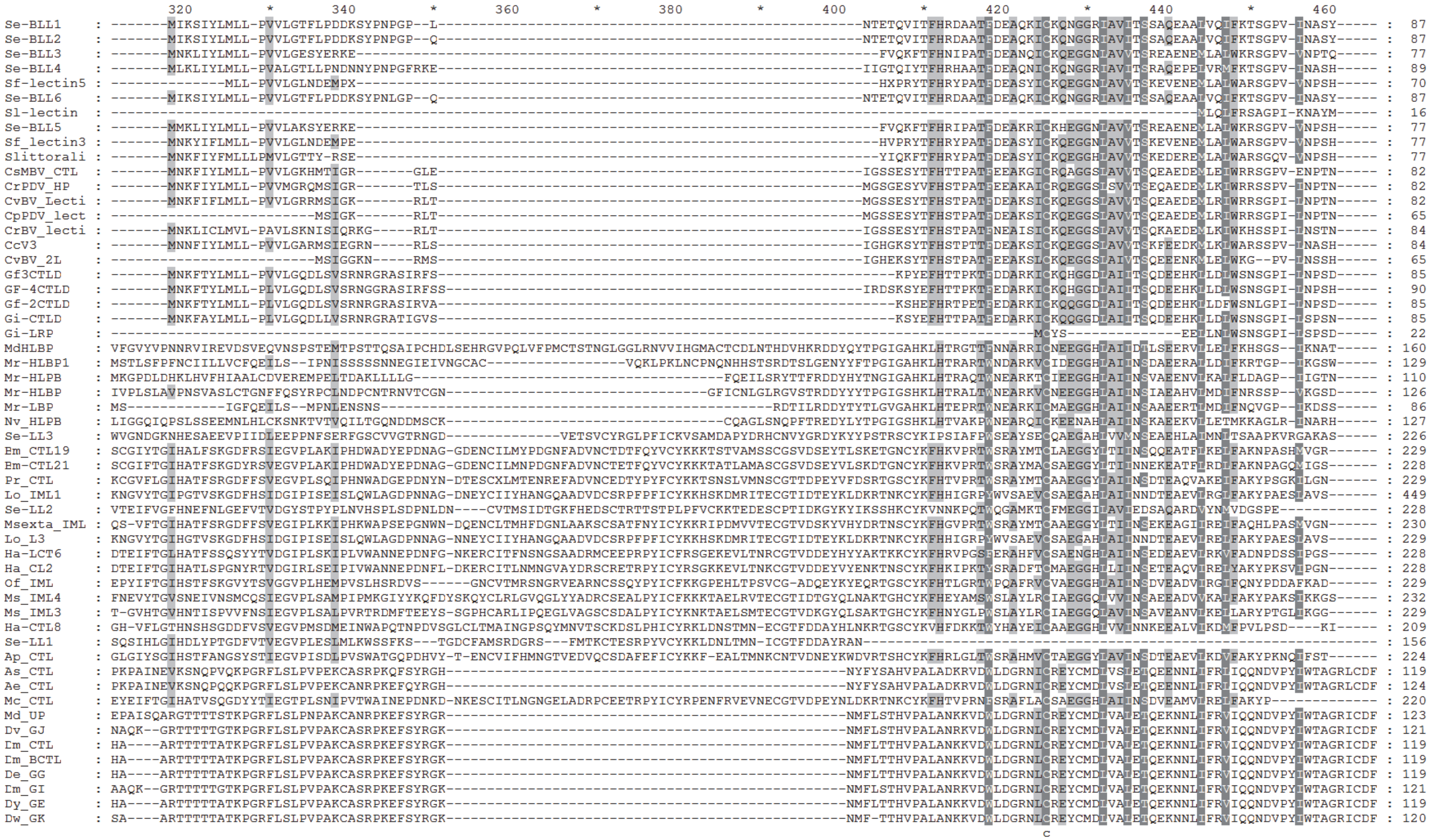

B (continuation)

```
                 *        480        *        500        *        520        *        540        *        560        *        580        *        600
Se-BLL1    : ----GWNLQAFIGIQ-YSQSGQWQTLDGK-------PAPYLNWSR------NWMG--VQPSNPTACRCGSILL-----QGAMDDVQCD-VKLAFFCEKVQKKSPTL-------------------------------------- : 167
Se-BLL2    : ----GWNLQAFIGIQ-YSQSGQWQTLDGK-------PAPYLNWSR------NWMG--VQPSNPTSCRCGSILL-----QGGMDDVQCD-VKLAFFCEKVQKKVQPCDCDV---------------------------------- : 171
Se-BLL3    : ----GLNAQAFIGIQ-LTSKG-WQTYFGE-------NPPYFNWSS------TWCGH-QQPDNPAMCKCGSILK-----QGGMDDVECH-YKLAFFCENWKVQNTDRHNLSNKCSTYD--------------------------- : 168
Se-BLL4    : ----GWNRQAFIGIQ-YRGSGLWQTLDGK-------PAPYLNWSR------NWMG--VQPSNPDRCRCGSILL-----QGAMDDVQCG-VRLAFFCEKVEEKLIPCNCSF---------------------------------- : 173
Sf-lectin5 : ----GLNAQAFIGLQ-LTSYG-WKTIYGR-------YPPYLNWSS------TWWGR-QQPDSPAYCKCGSILR-----QGGMDDVHCY-YKLAFFCEN---------------------------------------------- : 142
Se-BLL6    : ----GWNLQAFIGIQ-YSQSGQWQTLDGK-------PAPYLNWSR------NAVVCCYKEAWTMCSVTSNSLS-----SAKKYRKKSS-LVIAMFRFRFFLNIV---------------------------------------- : 167
Sl-lectin  : ----GWDQQAFIGMH-YS-NGKWLTLDGK-------PAPYINWST------TWYG--GQPSNPVWCSCGSILK-----QGAMDDVQCN-VKLAFFCEKT--------------------------------------------- :  88
Se-BLL5    : ----GLNAQAFIGIE-LTSKG-WQTTFGD-------RPPYLNWSL------NWWGR-RQPDSPTICKCGSILK-----QGGMDDVHCY-YKLAFFCENWKAQNTDRRNFSNKYSLHSVFCGFLKDFFSNNINFFVSYIVFHEFC : 195
Sf_lectin3 : ----GLNAQAFIGLQ-LTSYG-WKTIYGR-------YPPYLNWSS------TWWGR-QQPDSPAYCKCGSILR-----QGGMDDVHCY-YKLAFFCEN---------------------------------------------- : 149
Slittorali : ----GLNAQAFIGLQ-LSSRG-WQTVFGG-------YPPYLNWSS------NWWGR-QQPDNPTICKCGSILK-----QGGMDDVHCY-YKLAFFCGKLASPHYW--------------------------------------- : 156
CsMBV_CTL  : ----GLGSQAFIGIHSLNVKGHWETIDGE-------SPKYINWSQ------DWAGG-RQPSSPSVCKCGSILK-----QGRMDDVECF-FKLGFFCTKN--------------------------------------------- : 157
CrPDV_HP   : ----GLVSQVWIGIHSLNQEGHWETIDGE-------SPRYMNWSK------DWAGG-PQPSTPSVCKCGSILK-----QGGMDDVECF-FKLGFFCTRA--------------------------------------------- : 157
CvBV_Lecti : ----GLKLQAYIGIHSLNKEGHWETIDGE-------SPRYINWSQ------DWAGG-RQPSTPSVCKCGSILK-----QGGMDDVECY-FKLAFF------------------------------------------------- : 153
CpPDV_lect : ----GLKLQAYIGIHSLNKEGHWETIDGE-------SPRYINWSQ------NWAGG-RQPSTPSVCKCGSILK-----QGGMDDVECY-FKLAFFCTSV--------------------------------------------- : 140
CrBV_lecti : ----GLTSQAFIGIHSLNKKGHWETIDGE-------SPKYINWSQ------HWSGG-RKPSTSSVCKCGSILK-----HGGLDNVECY-FKLAFFCTKE--------------------------------------------- : 159
CcV3       : ----GFNHQAFIGIHSLNEEGHWETINGN-------SPSYVNWSP------NWTGN-QQPDNSSKCKCGSILK-----QGGMDDIECH-LKIAFFCEKLK-------------------------------------------- : 160
CvBV_2L    : ----GYNYQAFIGIHSLNKEGHWETIDGS-------SPKYVNWSP------DWTGR-QQPDTPSKCKCGSILK-----QGGMDDIECY-LKIAFFCEK---------------------------------------------- : 139
Gf3CTLD    : ----GLDKQVFIGVNNLQDVDRWETIKGE-------PLPYDNWSS------WWANG-RQPSRPKECRCGSILR-----QGGMDDVECY-LKLGFICESS--------------------------------------------- : 160
GF-4CTLD   : ----GLDKQVFIGVNNLQDVDRWETIKGE-------PLPYDNWSS------WWANG-RQPSRPKECRCGSILR-----QGGMDDVECY-LKLGFICESS--------------------------------------------- : 165
Gf-2CTLD   : ----GLDKQVFIGVNNLRNVDRWETIKGE-------PLPYENWSS------WWAGG-RQPSRPKECRCGSILR-----QGGMDDVECY-LKLGFICESS--------------------------------------------- : 160
Gi-CTLD    : ----GYDKQVFIGVNNLRDVNRWETIEGE-------SLPYDNWSS------WWADG-RQPSRPNECRCGSILR-----QGGMDDVECY-LKLGFICESS--------------------------------------------- : 160
Gi-LRP     : ----GLDKQVFIGVNNLRDVNRWETIEGE-------SLPYDNWSS------WWAGG-RQPSRPNECRCGSILR-----QGGMDDVECY-LKLGFICESS--------------------------------------------- :  97
MdHLBP     : -----NEDQAFIGIHDLFSEGEWVTILGES---L-HKYGYTKWSD------RWGG--QPDNGGGVCNCGSILK-----EGGMDDVSCN-AQFAFFCEIPEIRIAH--------------------------------------- : 242
Mr-HLBP1   : -----VNDEAFLGIHDLFAEGDWTTVFGDS---L-AKAGFTTWSN------KWSG--QPDNWNNTCHCGAILK-----EGGMDDVTCN-QPHAFFCELPNILFLNQDIS----------------------------------- : 215
Mr-HLPB    : -----DDYEAFIGIHDQFAEGDWTTVHGDS---L-SKTGFTKWSD------RWNG--QPDNWSSKCHCGAILR-----EGGMDDTYCY-KPFAFFCELPVSS------------------------------------------ : 189
Mr-HLBP    : -----VTGQAYIGTHDLYAEGDWTTILGDS---L-AKTGYTEWSD------KYNG--QPDNTGGVCNCGSIFR-----DGKMDDIRCD-LPHAFFCELSCSSLL---------------------------------------- : 207
Mr-LBP     : -----INDEALLGIHDQFVEGEWITLFGDP---L-EQTGYSKWSD------KWGG--QPDNGYNASNCGAILK-----EGGMDDVSCK-LPYGFFCELP-I------------------------------------------- : 163
Nv_HLPB    : -----TTDQAYLGIHDFFKEGEWLTIFGES---I-YSAGYAEWST------NWYVR-QPDNAGNGENCGTLVL-----NGGMNDNACH-IPLAFFCETPLPCTD---------------------------------------- : 209
Se-LL3     : -------YFFFAGFRAEKPVGNATVVFKTIFNETLSQAGYENWSD------------NEPNNSNGEYCGSIFLN----DGKLNDIHCH-DTFAFICEKEVSS------------------------------------------ : 304
Bm_CTL19   : ----FWKDIAFIGLHDWNEHGEWL-TINGET--L-PEAGYDKWSA------GEPNNSTGG-----EYCGSIYR-----SALINDIWCG-RPAPFICEKEPRS-LLREHDDK--------------------------------- : 314
Bm-CTL21   : ----FWKDVAFIGFHDWNERGEWL-TINGEK--L-QEAGYEKWSG------GEPSNATTG-----EYCGSIYR-----SALLNDIWCE-KPAPFICEKEPRS-LLREHDDK--------------------------------- : 313
Pr_CTL     : ----YWKDVAFIGFHDWGEHGEWL-TIISDT--L-QEAGYSKFSG------GEPNNATTG-----EFCGAIYR-----NGMFDDIWCE-NRYAFFCEKDPGS-LMCEQDE---------------------------------- : 313
Lo_IML1    : ----Y-HDAIRLGFYNWNDESNYFGTLHGQS--L-KEAGYEKWAR------NQP-TFHHGGSP--QKCGGMFR-----SALFDDTNCE-DNLAFVCEKDPESLVSFNLDNRYGDN----------------------------- : 541
Se-LL2     : ---------YFVGVRRLFSRSDYYTVKGHKFS-DMYSLSYNDQNG---------------------DCGTVSSR--NSDRIYTSTADCDQRLPFICEMEAQK------------------------------------------ : 297
Msexta_IML : ----FWKDMAFVGFHDWGEHGTWL-TVQGQT--L-EEAGYAKFAP------GEPNNATTG-----EYCGGVYR-----TGLLDDIWCE-NVYAFICEKDPNS-LLCDPTSDSFDDIIDIRNVN--------------------- : 327
Lo_L3      : ----Y-HDAIRLGFYNWNDESNYFGTLHGQS--L-KEAGYEKWAR------NQP-TFHHGGSP--QKCGGMFR-----SALFDDTNCE-DNLAFVCEKDPESLVSFNLDNRYGDN----------------------------- : 321
Ha-LCT6    : ----FRKEVAYIGFHDWGTWGDFR-TVHGET--L-KEAGYDKFSG------GEPNNATTG-----EHCGAIYR-----SALLDDIWCD-KPVPFICEKDPAYPPICQPTADEEIDIDERFNNKVE------------------- : 328
Ha_CL2     : ----FDKDKAFIGFRAVDEWGDWR-TVHGET--L-HEAGYDLFSS------GEPNNRTAG-----QYCGGIYR-----NALLDDMWCE-TQYVFICEKDPEYPPVCQPSKDEEGQ----------------------------- : 318
Of_IML     : ----A-KYAASIGFQGWGAVKIWW-TIHGQT--L-QDAGYSKWDK------FVPDMRSTR-----HYCGAVGR-----NGTLSHIECEGVTFPAICEKKADLV----------------------------------------- : 307
Ms_IML4    : ----EPVNVIFVGFRDLNQSNVWR-TINGQS--L-EEAGYANWAA------GEPNNVINQK----QYHGAMYR-----EGGLD-DYNHDVPAPFICEKHPHNIVPVPNNG---------------------------------- : 318
Ms_IML3    : -----YAGGAFLGFHDWNNNNVWR-TVNGQT--L-EEAGYANWGV------TQPDSSVQN-------CGQMFR-----SGQLD-DIG-CAKVPFICEKHPNNIMPVPNNV---------------------------------- : 310
Ha-CTL8    : ----NKWEQFHIGMRAYGNQRTWV-TLNGDR--L-EDV-FNDWDP------GQPDNMSGK-----QHHATFIR-----AGTLDDGNSE-TKAKFVCEKSPKVELFEPSPLQYSEISGKAERY---------------------- : 305
Se-LL1     : -----DWENFHAGLRACGPERTWI-TIHGER--I-EDV-FHDWNP------GQPDNYMGI-----QNSGSFIR-----LGTLDDHASD-KKSMFVCEKDPKVKRFEEVPEDCRSFRAILDRKLFLNPFT--------------- : 258
Ap_CTL     : ----Y-QDSAVIGFREWPEERSWY-TVHGQT--L-EDAGYDKWPT------GQPDNAYLGGET--QSCGAIWR-----TALLDDVWCN-QQFAFICEKDPQ-----YSPNIY-------------------------------- : 308
As_CTL     : KGCEGRPDLEPKNIYGWFWSNNREKIHPTNQ--IPNGWGYNPWSKSGHKKIPQPDNAEFDINQTTESCLSILNNVYNDGIGWHDVACY-HEKPVVCEDS--------------------------------------------- : 215
Ae_CTL     : KGCENRPDLEPKNVYGWFWSNNREKIPATNK--IPNGWGYNPWSKSGHKKIPQPDNAEFDINQTTESCLSVLNNVYNDGIGWHDVACY-HEKPVVCEDSEELLNYVAATNPGIRL----------------------------- : 236
Mc_CTL     : ------------------------------------------------------------------------------------------------------------------------------------------------ :   -
Md_UP      : NGCENRPDLEPKNIYGWFWSATREKIQATNR--IPQGWGYNPWSQTGHKKRPQPDNAEYDINQTKEQCLSVLNNVYNDGIAWHDVACY-HEKPFICEDNDELLRYVAATNPGIRL----------------------------- : 235
Dv_GJ      : AGCENRPDLEPKHVYGWFWSATREKIQATNR--IPQGWGYNPWSQTGHKKRPQPDNAEYDINQTKEQCLSVLNNVYNDGIAWHDVACY-HEKPVICEDNEELLRYVAATNPGIRL----------------------------- : 233
Im_CTL     : AGCENRPDLEPKTVYGWFWSATREKIQATNR--IPQGWGYNPWSQTGHKKRPQPDNAEYDINQTKEQCLSVLNNVYNDGIAWHDVACY-HEKPVICEDNEELLRYVAATNPGIRL----------------------------- : 231
Im_BCTL    : AGCENRPDLEPKTVYGWFWSATREKIQATNR--IPQGWGYNPWSQTGHKKRPQPDNAEYDINQTKEQCLSVLNNVYNDGIAWHDVACY-HEKPVICEDNEELLRYVAATNPGIRL----------------------------- : 231
De_GG      : AGCENRPDLEPKTVYGWFWSATREKIQATNR--IPQGWGYNPWSQTGHKKRPQPDNAEYDINQTKEQCLSVLNNVYNDGIAWHDVACY-HEKPVICEDNEELLRYVAATNPGIRL----------------------------- : 231
Im_GI      : AGCENRPDLEPKNIYGWFWSATREKIQATNR--IPQGWGYNPWSQTGHKKRPQPDNAEYDINQTKEQCLSVLNNVYNDGIAWHDVACY-HEKPVICEDNEELLRYVAATNPGIRL----------------------------- : 233
Dy_GE      : AGCENRPDLEPKTVYGWFWSATREKIQATNR--IPQGWGYNPWSQTGHKKRPQPDNAEYDINQTKEQCLSVLNNVYNDGIAWHDVACY-HEKPVICEDNEELLRYVAATNPGIRL----------------------------- : 231
Dw_GK      : AGCENRPDLEPKNVYGWFWSATREKIQATNR--IPQGWGYNPWSQTGHKKRPQPDNAEYDINQTKEQCLSVLNNVYNDGIAWHDVACY-HEKPVICEDNEELLRYVAATNPGIRL----------------------------- : 232
                         g                              s                       c                  c        ce
```
