# Supplementary material for: Recurrent Domestication by Lepidoptera of Genes from Their Parasites Mediated by Bracoviruses
Source: PLoS Genet. 2015 Sep 17;11(9):e1005470. doi: 10.1371/journal.pgen.1005470 (PMC4574769; doi:10.1371/journal.pgen.1005470)
Supplement: S1 Table — (DOCX) [file pgen.1005470.s007.docx]

Table S1. Primers used in the study

| Primer name | Sequence (5´-3´) | Application |
| --- | --- | --- |
| **Ben9 12F** | GGTGACAGCGTTTCTTTCGT | Ben9 insertions in *D. plexippus* |
| **Ben9 12R** | GTCCCTTCCAGTCCGTTGTA | Ben9 insertions in *D.* *plexippus* |
| **Ben9 13F** | ATTTTGTCCGATGGATGGAG | Ben9 insertion in *D. chrysippus* |
| **Ben9 13R** | GATCAGTGCCGACTCAACAA | Ben9 insertion in *D. chrysippus* |
| **Ben9 EXF** | GCAGATTCGATTGGTTGGTT | Ben9 mRNA Expression |
| **Ben9 EXR** | CGTTTTCCGTCGTTCAGTTC | Ben9 mRNA Expression |
| **EF1αF** | ATTGGTACAGTGCCCGTAGG | DNA sample quality |
| **EF1αR** | GCGTGTAACCGTTGGAGATT | DNA sample quality |
| **5p-BV2-5** | TTCATGCTCCTCTGCTTGTATCCTCG | Genome walking (BV2-5) |
| **5s-BV2-5** | GCAACGTTAGTATGGTAATAGGCAACA | Genome walking (BV2-5) |
| **3p-BV2-5** | CTTATTTGTCCGTTAGGTTTGGTGTGCTTG | Genome walking (BV2-5) |
| **3s- BV2-5** | GGTGATGGTGATGGTGATACTGGAATTGG | Genome walking (BV2-5) |
| **5p-Se-BLL2** | CTGGGCTTCATCAAATGTTGCAGCAT | Genome walking (BLL2) |
| **5s-Se-BLL2** | GTTCTGAGGTCCTGGATTCGGGTAGCTT | Genome walking (BLL2) |
| **3p-Se-BLL2** | CAAATCAGTCTTGCCGTTTTTGGCTTGA | Genome walking (BLL2) |
| **3s-Se-BLL2** | GATTTTCAGAGTAGCCACAGAGTGTTAAG | Genome walking (BLL2) |
| **BV2-5ORF-F** | CGTGCGAGTTGAAAGCATAG | Sequence amplification of BV2-5 in different populations of *S. exigua* |
| **BV2-5ORF-R** | TCCACTGAGTTCCGCTTTTAG | Sequence amplification of BV2-5 in different populations of *S. exigua* |
| **BV2-5Ac-F** | ACTCGAGATGTTGCCTATTACCATACTAACG | BV2-5 Cloning in AcMNPV |
| **BV2-5 Ac-R** | TATGCATTTAATGGTGATGGTGATGGTGATACT  GGAATTGGACATATTTGAGC | BV2-5 Cloning in AcMNPV |
